# Modelling Seeded Growth of Nanoparticles Far from Equilibrium

**DOI:** 10.3390/nano16211383

**Published:** 2026-10-28

**Authors:** Gilberto Nardi, Sofia Zinzani, Leonardo Stefanelli, Francesca Baletto

**Affiliations:** Physics Department, Univeristà degli Studi di Milano, Via Celoria 16, 20133 Milano, Italy; gilberto.nardi@unimi.it (G.N.); leostefa002@gmail.com (S.Z.); leonardo.stefanelli2@studenti.unimi.it (L.S.)

**Keywords:** metallic nanoparticles, formation processes, molecular dynamics, pySNOW

## Abstract

As modelling the synthesis of metallic nanoparticles leads to useful insights that guide experiments, we propose a novel numerical suite, NPGrowth, which is based on LAMMPS. NPGrowth enables us to simulate seed-mediated growth under a variety of conditions, even mimicking growth under extreme conditions. It allows one-by-one or N-by-N deposition, either when the incoming atoms are from uniformly distributed or well-defined and fixed spatial regions, onto selected targets, including those reminiscent of a chiral molecule. Moreover, NPGrowth enables the formation, through sintering, of two or more seeds, be that directional, soft-landed or through coalescence. After presenting a few toy models, we discuss the effect of directionality during the sintering of four Cu icosahedral seeds and the importance of identifying the sintering geometry following a pySNOW characterisation and classification.

## 1. Introduction

As synthesis is the art of manipulating atoms at the nanoscale, a fundamental understanding of nucleation and growth mechanisms is essential to leverage the highly sensitive, morphology-dependent chemophysical properties of metallic nanoparticles [1,2,3,4,5]. Nanoparticle morphology, which encompasses size, shape, structural motif, and chemical ordering, is crucial for a range of applications, including catalysis, optoelectronics, biomedical applications and sensing. The appearance of a given nanoparticle morphology depends on our ability to engineer synthetic outcomes precisely [6,7,8,9,10,11]. As an example, in catalysis, tuning nanoparticle morphology can significantly influence their catalytic activity; for instance, a CO molecule cannot adsorb at mixed sites on AuRh nanoparticles [12], and oxygen’s reduction-reaction activity can be estimated from geometric considerations of nanoparticle adsorption sites [13,14,15,16].

Proposing a robust simulation tool to model metallic nanoparticle (MNP) formation requires detecting, analysing, and incorporating a variety of individual mechanisms that occur on different time scales and with different activation energies. The interplay of these subprocesses, spanning from quantum-level chemical bonds to macroscale fluid dynamics, shapes the final morphology of the nanomaterial. Because of this complexity, a multiscale modelling approach is needed. Mechanism-enabled population balance modelling accounts for not only primary phenomena such as nucleation and growth but also for particle agglomeration and breakage, with a focus on reproducing the particle size distribution. For sizes larger than 5 nm, the model presented in Ref. [17], based on classical nucleation theory and heterogeneous nucleation using the general aerosol dynamic equation, should be considered.

In the size range between 1 and 10 nm, classical Molecular Dynamics (cMD), which solves Newton’s laws of motion, enables the tracking of structural trajectories and elucidates structural transformations during synthesis across different timescales and temperatures. The resulting nanoparticle morphology can be compared with the putative global minimum, which defines the thermodynamic endpoint the cluster inherently drives toward. Comparisons are usually based on geometric descriptors. Bochicchio et al. [18] focus on global optimisation, thermodynamic stability, and chemical ordering layouts—such as core-shell, onion-like, or random-alloy nanoalloys. However, it was recently shown that far-from-equilibrium morphology can ensure better performance [2,19,20,21,22]. With recent developments in machine-learned potentials (MLPs) designed specifically for nanoparticles [23,24,25], the quantum description of 1–10 nm objects can be incorporated into the interparticle potential. Nonetheless, cMD applied to MNP growth relies heavily on semi-empirical potentials, such as tight-binding second-moment approximation potentials or the embedded-atom method (EAM), with a few cases being described by machine-learning potentials [26].

Among the several cMD studies reported in the last three decades following the pioneering works of Manninen [27]—inspired by surface-growth models—and Mariscal [28,29], Grammatikopoulos [30] and Baletto [31,32,33] proposed three growth routes onto an existing metallic seed: (i) one-by-one atomic deposition from random positions tuned by different deposition rates and temperatures; (ii) the coalescence of two or three seeds along their centre of mass axis; and (iii) the formation of a seed as obtained from the freezing of a nanodroplet. The effects of the relative orientation of facets, e.g., (111)/(111) and (111)/(100), have been widely investigated [26,34,35,36,37]. There is agreement that when the impact velocity or initial temperature is high enough, the kinetic energy supply allows surface restructuring, followed by reordering of atoms at the core. Zinzani et al. [38,39] recently showed that the neck has a short lifetime of a few ps, at least for Au-based nanoalloys.

These computational approaches highlight the importance of kinetic trapping, out-of-equilibrium pathways, and structural transitions, and show how competition between flux-landing rates and surface-diffusion energy barriers leads to metastable, faceted geometries or amorphous traps. Their work details how distinct, preformed seeds collide in flight, undergoing structural rearrangements, rigid-body reorientation, eventual temporary interfacial melting, neck formation and surface adatom diffusion to fill necks [30,31,37,38,39,40,41,42,43,44,45].

However, the main limitation of existing approaches is their neglect of directionality in atomic deposition and/or cluster sintering, and, thus, its failure to model seeded growth under certain conditions. While random deposition can mimic gas-phase formation, it may fail when the nanoparticles grow in wet environments where molecules can shadow certain regions of the growing seed, or during cluster-beam deposition.

To overcome these limits, we provide an open-source software tool, NPGrowth, that models metallic nanoparticle growth based on cMD and is embedded in LAMMPS [46]. We retained the LAMMPS core because it supports a wide range of interatomic potentials, including MLPs, EAM, and Rosato–Guillope–Legrande (RGL). NPGrowth allows deposition of individual atoms/clusters from one or more sources onto one or more designed targets. NPGrowth enables the formation of elongated, prolate, and even chiral metallic nanoparticles. NPGrowth also provides tools to simulate nanoparticle sintering and aggregation starting from an adaptable initial configuration. By enabling atomistic simulation of nanoparticle formation under a wide range of user-defined conditions, NPGrowth supports the design and comparison of experimental synthesis strategies while letting computational researchers explore standard and unconventional growth pathways and nanostructures with potentially novel physicochemical properties. We further discuss, in detail, the dynamics of four Cu seeds during directional sintering. To highlight the morphological differences, we employ a recently developed general characterisation tool, pySNOW [47,48,49]. Building on recent developments in morphological descriptors [26,49,50], we introduce a nanoparticle ID that reports a small set of geometric descriptors for identifying and classifying nanoparticles, and we hope it is flexible enough to interest the community.

## 2. Methodology

NPGrowth is a lightweight, open-source code that enables workflows by coupling a declarative description of the deposition/sintering geometry with a modified version of the fix deposit command in LAMMPS. The code is openly available at https://github.com/nanoMLMS/NPGrowth_V2, (accessed on 22 September 2026) together with example input files. The Appendix A provides an overview of the code and its use. To provide a consistent framework for describing the different growth scenarios, we summarise the terminology adopted throughout this work in Box 1.

Box 1 **NPGrowth-related dictionary and variables adopted in this work.**

- Initial seed: a MNP placed in the simulation box.
- Source region(s): NPGrowth identifies each deposition source through an insertion region in absolute simulation-box coordinates. The source region(s) (SRs) are the volume where atoms or other seeds are generated and from which move towards the seed. Multiple sources can be combined to realize complex deposition geometries.
- Target region(s): NPGrowth directs each deposited particle toward a user-defined target region. A target region is built as the union of one or more axis-aligned cuboids and can represent anything from a near-point target (e.g., the cluster’s centre of mass) to an extended or multi-lobed region, allowing, for instance, a beam to be split with equal probability between several distinct impact sites.
- Deposition mode: NPGrowth allows atom deposition or sintering. Atoms can be deposited from random or fixed directions. Either 1-by-1 (single) or N-by-N (multiple) atoms are deposited after every step, with every being a fixed integer number, from each source region.
  For sintering, we choose the *sintering geometry* as how the seeds are initially arranged. NPGrowth offers scripts to produce initial configurations for sintering simulations involving two or more seeds by specifying the desired geometrical arrangement (e.g., line, triangle, square, or tetrahedron) and the seed displacement in space, with MNPs oriented randomly. This allows us to thoroughly investigate the role and effects of the number, initial geometry, relative positions and orientations of the seeds.
- Deposition rate (*ρ*): number of atoms deposited every “every” MD integration timesteps (*δt*): *ρ* = *N*/(every·*δt*), where every is an integer number.
- Deposition kinetic energy (or source temperature): the speed of the injected atoms/clusters with respect to the initial seed. In the soft-landing regime, we consider the kinetic term of the deposited atom(s)/nanoparticle as negligible.

**Figure 1:**
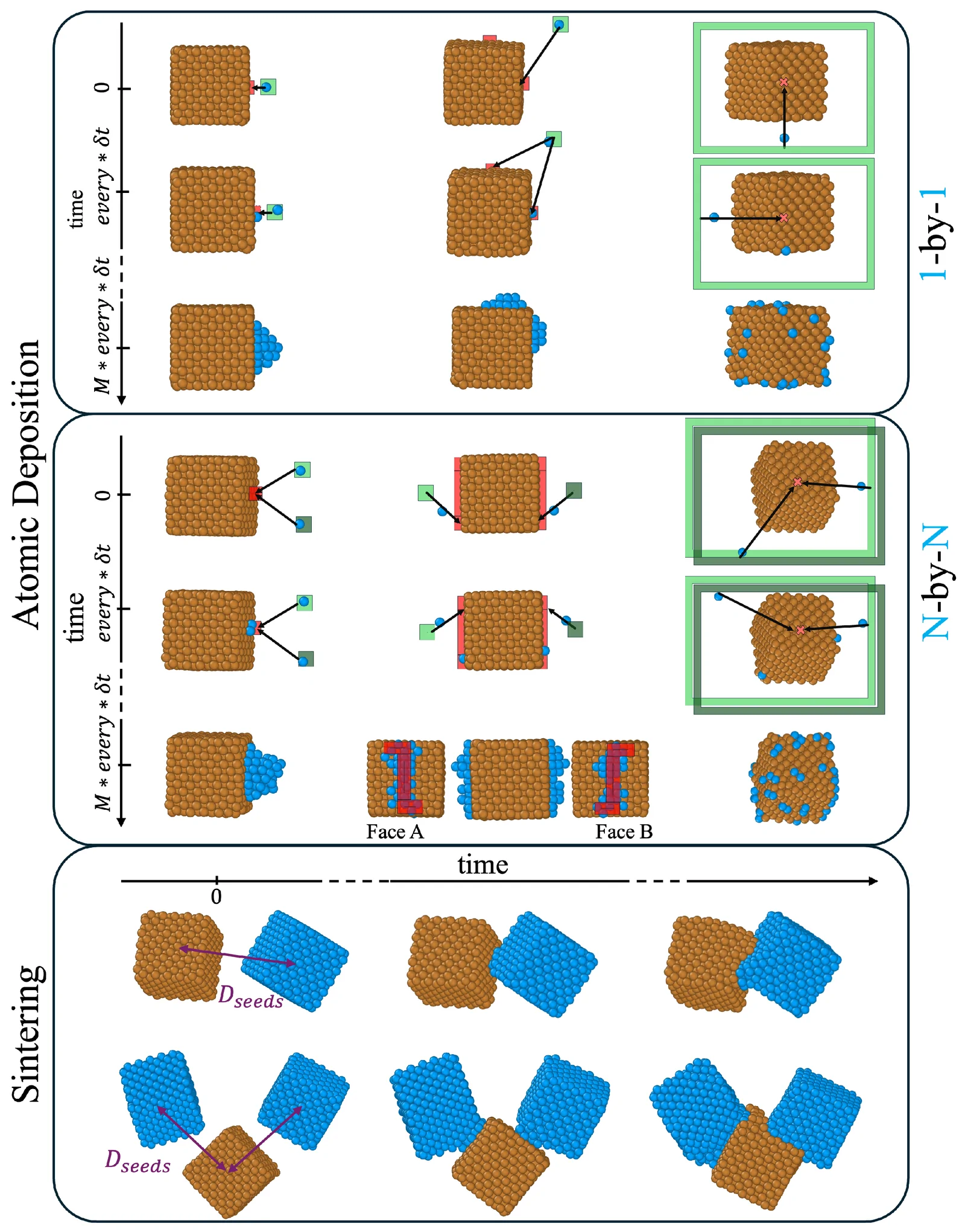
Pictorial representation of processes simulated by NPGrowth. **1-by-1 atomic deposition**: as time progresses at each *every* step, atoms are deposited from (left column) one fixed source to a fixed target region; (middle) one fixed source towards two fixed target regions; and (right) randomly selected sources toward the seed centre of mass (CoM). **N-by-N atomic deposition:** *N* atoms are deposited simultaneously at each deposition event, with 2-by-2 coming from (left column) two fixed sources onto a fixed target region; (middle) two fixed sources onto a target region with an S-geometry; and (right) from randomly selected positions toward the CoM. Source regions are in green and target regions in red, with red crosses indicating the CoM. Arrows connecting the source and target regions are the deposition trajectories. Deposited atoms are shown in blue, while the seed atoms are in orange. **Sintering stages:** (upper) two Cu cubes positioned initially at $D_{\mathrm{seeds}}$ and approaching along the line connecting their CoM and (bottom) three Cu cubes initially in a triangular geometry.

As depicted in Figure 1, NPGrowth automates the modelling of seed-mediated growth, enabling the modelling of the formation of metallic nanoparticles even under extreme conditions, i.e., chemical supersaturation, where any corner or vertex acts like an exposed peak [8,9,37,51], leading to branched and chiral structures too.

Simulating MNP growth by atomic deposition requires a standard molecular dynamics setup and a deposition protocol that specifies the deposition parameters. NPGrowth keeps the cMD setup and the deposition setup fully decoupled, with LAMMPS handling the cMD engine. The user writes the cMD setup as an ordinary LAMMPS input script. The deposition setup is described declaratively in JSON files and compiled by a Python 3.12 script, generate_depo_input.py, into a LAMMPS include file containing the corresponding source and target regions, and the fix deposit commands. This separation lets the user reuse the same deposition configuration across different potentials, thermostats, or seed geometries without modification.

Related to the deposition protocol, LAMMPS’s native fix deposit inserts atoms with a velocity sampled independently along each Cartesian axis, which does not allow a beam to be aimed at a chosen location on an arbitrarily shaped, off-centre seed cluster. When a highly directed, collimated atomic beams hits the nanoseed, mimicking a Glancing Angle Deposition or any experimental setups with preferential direction, atoms preferentially land on the “windward” sides of the seed, leading to surface bumps (in a highly non-equilibrium kinetic regime governed by ballistic shadowing), forcing an isotropic seed into an elongated nanoparticle, nanoneedle or even a nanorod [52,53]. The key methodological novelty of NPGrowth is a pre-processing modification of LAMMPS fix_deposit.cpp. To allow for the deposition of atoms/clusters/molecules from an arbitrary source region to another arbitrary target region, the LAMMPS fix deposit command has been modified to accept a region ID as a target. This overcomes the limitation of the original fix deposit command, which allows us to specify a range of velocities or a single target position per command, which is not enough to describe all possible deposition geometries. At each insertion attempt, a candidate position is drawn inside the source region (subject to a minimum-distance constraint from existing atoms), a point is drawn uniformly inside the associated target region, and the velocity direction of the inserted particle is set to point from the former to the latter. In contrast, its magnitude is drawn independently from a user-specified speed range. This makes the deposition direction a direct, geometric consequence of the relative placement of both the source and target regions. Atoms are classified by their coordination number and assigned to the growing seed. Only atoms that have become part of the growing seed are coupled to the thermostat. In contrast, newly deposited atoms evolve under plain NVE dynamics until they attach, mimicking the physically distinct regimes of an incoming versus an incorporated atom.

### Nanoparticle Characterisation Using pySNOW

We employ our recently developed pySNOW—a Python Suite for the NanO-World [48]—to characterise the morphology of MNPs. pySNOW is an agile and open-access numerical tool that reads nanoparticles’ trajectories/position and calculates various geometrical descriptors, i.e., the maximum pair distance, the maximum radial distance, the radius of gyration, and the relative shape anisotropy. Structural descriptors include the common neighbour analysis (CNA) [5,42,54], and local atomic environment descriptors such as the atop generalized coordination number (aGCN) [55,56] and the CNA patterns (CNAPs) [48,49,57].

As in Refs. [49,58], the pair distance distribution function (PDDF) is crucial in determining the architecture of a MNP. Indeed, the PDDF provides the following: (i) its first minimum is the best estimate of the cutoff radius to identify the nearest neighbours; (ii) if the position of its second peak does not correspond to the lattice parameter, the structure is amorphous; and (iii) the maximum pair-atomic distance, $D_{max}$, is an estimate of the nanoparticle size. In addition, the shape of the PDDF relates to the shape of the nanoparticle [58,59]. In general, the shape and sharpness of subsequent peaks and minima highlight the degree of ordering of the structure over the range of distances considered.

Another robust global descriptor is the radial atomic density (RAD), which counts atoms in concentric shells around the nanoparticle’s centre of mass (CoM). Twice the maximum distance of RAD, $2R_{max}$, provides an estimate of the dimension of the whole MNP as it defines the sphere needed to include the whole nanoparticle. Moreover, integrating the RAD gives the number of atoms within a sphere of radius *r*, $N\left(r\right)=\int_{0}^{R}RAD\;dr$. We introduce the branching factor *f* by fitting:

(1) $$N\left(r\right)\propto r^{f}\;.$$

Completely packed spheres have f≈3, while branched or dendritic structures have f≲2.3. *f* values close in between show the formation of grooves and re-entrances or flattened/oblate shapes.

The radius of gyration of a rigid body is defined as the radial distance from a point having a moment of inertia equal to the effective mass distribution of the body if the total mass of the body is concentrated at that point. One can refer to the radius of gyration of a nanoparticle, i.e., the measure of the extent by which atoms are spread away from the centre of mass (${\mathrm{CoM}}_{w}$) of the nanoparticle.

$$G=\sqrt{\frac{1}{N}\sum_{i=1}^{N}{\left(r_{i}-r_{\mathrm{CoM}}w\right)}^{2}}.$$

We also use the gyration radius variation over time

(2) ΔG=G(t)−G(0).

to quantify distortion relative to the atom initial distribution. In particular, during sintering simulations we compute the per-seed value of ΔG to investigate seed-specific, geometry-dependent distortions during the processes. In this case, the sum in Equation (2) runs over the number of atoms in each seed, $N=N_{seed}$.

The $\alpha =G/R_{max}$ ratio gives information regarding the sphericity of the nanoparticle, with spheres featuring α→ 0.63, while elongated/rod-like shapes show α below 0.58.

Several global shape descriptors derive from the gyration tensor, $S_{mn}$:

(3) $$S_{mn}=\frac{1}{N}\sum_{i=1}^{N}r_{i}^{\left(m\right)}r_{i}^{\left(n\right)}$$

where $r_{i}^{\left(\gamma \right)}$ is the γ−th Cartesian component of the position-vector of the *i*-th atom, and m,n∈1,2,3. Among them, the relative shape anisotropy $\kappa^{2}$,

(4) $$\kappa^{2}=\frac{3}{2}\frac{\lambda_{1}^{2}+\lambda_{2}^{2}+\lambda_{3}^{2}}{{\left(\lambda_{1}+\lambda_{2}+\lambda_{3}\right)}^{2}}-\frac{1}{2},$$

where the $\lambda_{i}$ are the eigenvalues of *S*,which is a robust shape descriptor. $\kappa^{2}$ is null for spheres and Platonic solids, such as icosahedra (Ih) or cubes (Cb). While spheroid shapes have $\kappa^{2}≲0.01$, increasing $\kappa^{2}$ up to 0.25 identifies progressively more prolate and oblate structures. When $\kappa^{2}$ is above 0.25, structures become significantly prolate, such as nanorods, nanochains or nanotubes. We can further classify MNPs’ shapes as branched/dendritic (f≲2.3), compact (f>2.3).

We perform a CNA and CNAP to classify a MNP into geometrical families. CNA labels each pair of nearest neighbours from the local connectivity of their common neighbours. Each pair exhibits a signature, a triplet of integers, (r,s,t)- where *r* is the number of nearest-neighbours common to both atoms; *s* is the number of bonds between shared neighbours; and *t* is the longest chain which can be made from bonding s atoms if they are nearest neighbours-. The CNAP of an atom *i* is the string $\left(f_{1}{\left[r,s,t\right]}_{1};f_{2}{\left[r,s,t\right]}_{2};\ldots ;f_{k}\left[r,s,t\right]k\right)$ containing all the k−th CNA-signatures [r,s,t]k*i* is in. We note that the frequency of each signature correlates with the nominal coordination of the atom *i*, $\sum_{k}f_{k}\left(i\right)=CN\left(i\right)$.

Here, we consider FCC, twin FCC, and multiple-twinned nanoparticles [3] as decahedral (Dh) and Ih. Counting how many pairs contribute to selected CNA signatures provides information on the overall nanoparticle’s structure: FCC requires a zero (5,5,5) and a zero (4,2,2), while twin FCC has a non-zero (4,2,2). Multiple-twinned NPs have a positive (5,5,5) and a high (4,2,2). The FCC inner core can contain other defects, with a significant (4,1,1) component, mainly during the sintering process. Surface faceting follows: (111) facets feature a high (3,1,1), while (2,1,1) labels (100) facets. Distortions contribute as (3,2,2) for multiple-twinned shapes. To further distinguish decahedra from icosahedra, we consider the local environment of each atom through its CNA pattern. For example, following Ref. [49], [12(4,2,1)] is a FCC bulk; [6(4,2,1), 6(4,2,2)] labels atoms on a twin plane; and [10,(4,2,2), 2(5,5,5)] is those atoms lying on a fivefold axis. The [12,(5,5,5)] pattern refers to the Ih centre. On the other hand, a zero [12,(5,5,5)] with a high [10(4,2,2), 2(5,5,5)] stands for a Dh.

Besides the importance of associating atoms to their CNAPs, and each CNAP with specific atomic environments, we provide a short CNAP-summary as follows: (i) the number of different CNAPs, $N_{CNAP}$ and (ii) the number of singleton CNAPs -CNAPs appearing for only one atom. $N_{CNAP}$ decreases when the symmetry of the shape increases, being 1 in a perfect bulk, to $N_{CNAP}\to N$ for a completely disordered situation. Singleton CNAP, $U_{CNAP}$, provides a further estimate of the number of local defects, such as surface adatoms, islands, vacancy, and interstitial.

## 3. Results

**Figure 2:**
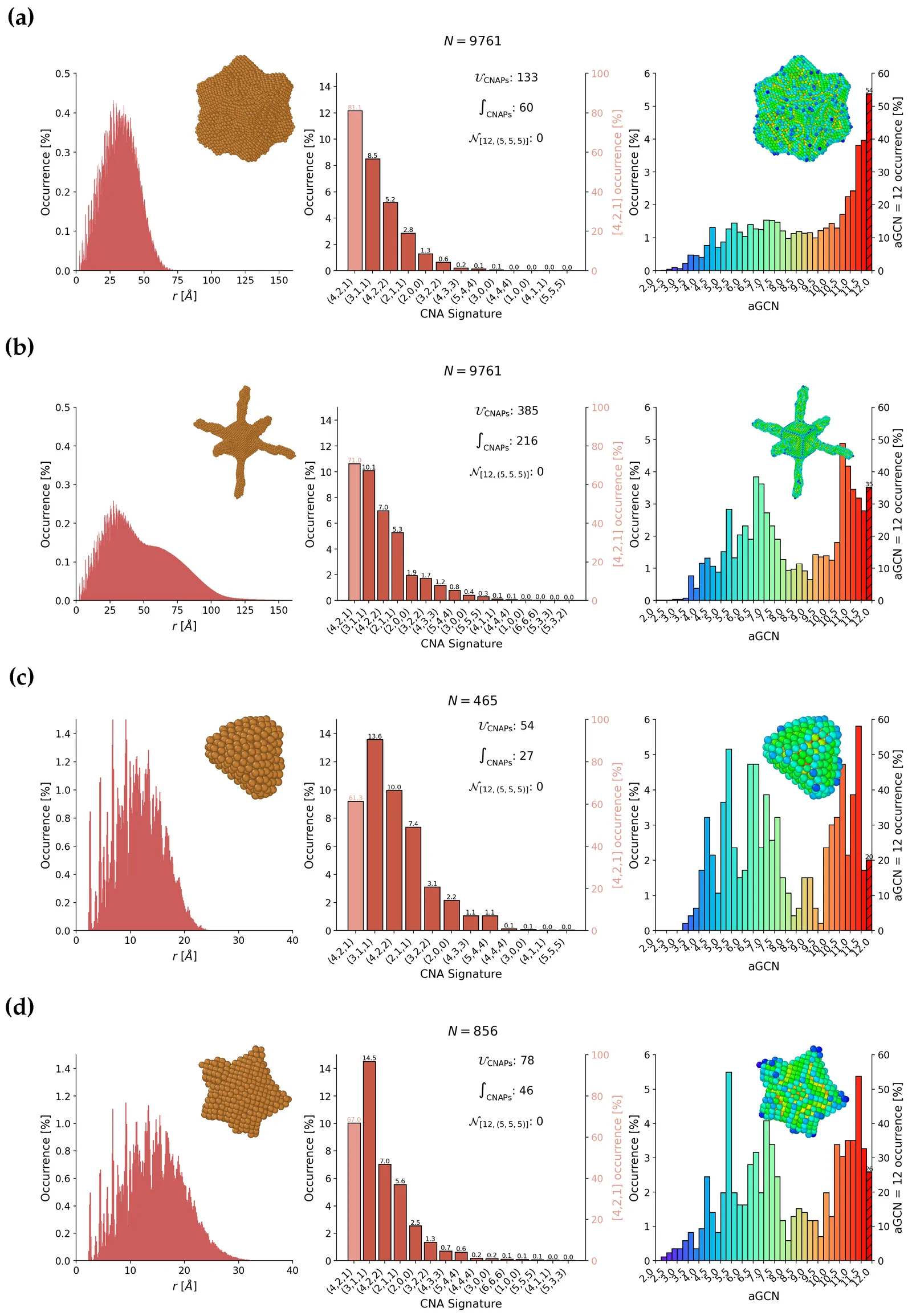
pySNOW characterization of last frame of Cu-NPs from the example listed in Table 1. (**a**) and (**b**) 5400 atoms deposited upon a Cb_4361_ and (**c**) and (**d**) atomic deposition upon a TO_201_ after 264 and 655 atoms, respectively. NP size as total number of atoms, *N*, at the top of each panel. From left to right: PDDF and snapshot of reference, with atoms coloured in dark orange; and CNA signature and CNAP summary. Values (4,2,1) occurrence (pink) to be read on the right axis-; atop-GCN distribution and relative snapshot with atoms coloured accordingly. The occurrence of aGCN = 12 (bulk environment) is tenfold and reported on the right axis. Representative snapshots are generated using OVITO  [60].

**Figure 3:**
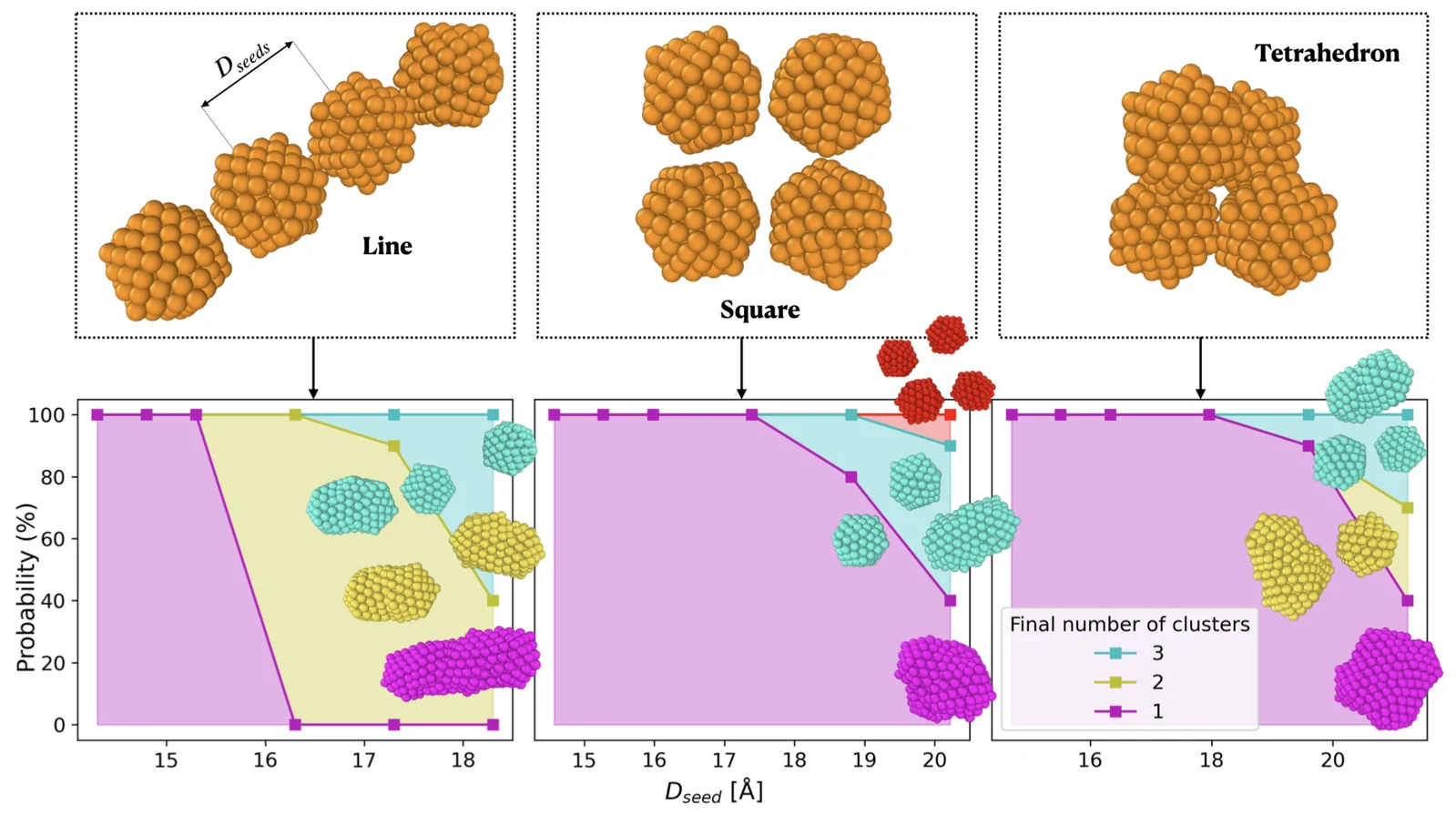
*Top row:* Sintering geometries with four seeds with respect to the relative position of their CoM, initially at a distance $D_{seed}$: anisotropic (Line); 2-D (Square); and a 3D isotropic arrangement (Tetrahedron). *Bottom Row:* Probability of forming a certain number of MNPs when four sinter in different initial geometries as a function of their relative displacement, $D_{seeds}$.

As the novelty of NPGrowth lies in its ability to mimic directional growth processes, we present a few examples, summarised in Table 1, that can lead to asymmetric, grooved or branched shapes. Although we do not pretend to reproduce any specific experimental situation, our examples would represent synthesis under far-from-equilibrium conditions. For the sake of completeness, we contrast this with random 1-by-1 and N-by-N atomic deposition; see Figure 2. We further consider the soft-landing of four Cu seeds from three different sintering geometries: (i) along a line, (ii) at the vertices of a square, and (iii) at the vertices of a tetrahedron. Figure 3 depicts these sintering geometries.

### 3.1. Directional Atomic Deposition on Cu-Seeds

We consider and discuss the four examples reported in Figure 2 and their shape classification, as shown in Table 1. The deposition rate and temperature are key parameters governing nanoparticle growth. These parameters tune the competition between atom attachment, adatom surface diffusion, collective and concerted mechanisms, and structural rearrangements. They, thus, affect the resulting morphology and enhance the possibility of kinetic trapping. Without claiming to mimic or reproduce any specific experimental regimes, they let us explore how imposed growth kinetics influence nanostructure formation. In contrast, a quantitative comparison with experimental growth processes would require simulations under more realistic conditions. Atomic deposition simulations are carried out using a timestep of 2 fs and a Langevin thermostat with a damping parameter of 0.1 ps. Interatomic interactions are described using the embedded-atom method (EAM) potential Cu_u3.eam, developed by Foiles et al. [61]. After each deposition event, the system evolves using a thermostat-coupled MD, allowing the deposited atoms and the initial seed to relax and evolve according to the imposed deposition conditions. Temperature is kept at 300 K.

Figure 2 reports the pySNOW analysis of the final snapshots of the four examples listed in Table 1. The final snapshots are (a) a concave cube, (b) a branched, spiky shape, (c) a flattened disk resembling an “onigiri,” and (d) a star shape with small re-entrances/grooves, but still classified as a spheroid.

The PDDF exhibits a second peak for all the structures, indicating a geometrical order. At the same time, we note that the PDDF profiles vary significantly. A branched shape leads to an almost flat region after reaching a peak. Grooves/re-entrances of star shapes are present but not too deep; the MNP retains a PDDF profile and a $\kappa^{2}$ value similar to that of compact shapes, although with less peaks at large distances. Moreover, the atop generalised coordination number (aGCN, [56]) distribution reveals a notably higher concentration of low-coordinated atoms, with an aGCN of below four, while flattened and compact shapes have clear peaks in correspondence with facet coordination and a drop in the number of atoms with an aGCN of around 8.5 and 10. Concavities of all sorts lead to a significant number of atoms with an aGCN of, mainly, around 10.

We note that branched MNPs, named *spike*, exhibit a wide variety of $N_{CNAP}$, with 56% being singletons and with 2% of atoms exhibiting an individual local environment, against the 0.6% from random deposition.

In all the examples, the combination of an initial FCC seed, a fast deposition rate and a low simulation temperature, show that the outcomes have a twinned FCC structure, with a zero (5,5,5), no sign of fivefold symmetries, and the (4,2,2) spanning from 5–10%, with the highest occurrence being on the *onigiri*. The CNAP analysis shows no Ih centres in any snapshot, as $N_{\left[12,(5,5,5)\right]}$ is zero. A common feature is the surface, with (3,1,1) being considerably higher in all snapshots, suggesting the formation of (111) facets and indicating that re-entrances have a (111) character, as the *star* has a (3,1,1) occurrence of around 14.5%.

### 3.2. Directional Sintering of Four Cu-Seeds

We soft-landed four Cu-Ih_147_ seeds from an equilibrated NVT MD trajectory. We ran simulations with a 2.5 fs timestep and a Langevin thermostat with a time-coupling constant of 100 timesteps. We modelled the system’s potential energy surface with a custom machine learning potential (MLP) built using the FLARE package [62,63]. Appendix B provides more details of the MLP derivation. We placed the studied sintering dynamics in the context of previous atomistic studies highlighting the roles of adatom surface diffusion, rearrangements of the whole nanoparticle, and twin planes formation/disappearance during nanoparticle sintering [30,64,65,66]. In addition, we point out that Cu nanoparticles can exhibit concerted rearrangements, up to a considerable size [67].

First, we carried out a series of short simulations to characterise the occurrence of four-way sintering as a function of the sintering geometry and of the initial displacement between the centre of mass of the seeds, $D_{seed}$, which spans a distance such that atoms from different seeds are in contact in such a way that they are at a minimum distance from the cutoff of the potential, where inter-seed interactions are negligible. For each of the three geometries, we performed 10 independent simulations over a range of initial displacement distances, using different random seeds that generate distinct initial cluster orientations. Each simulation runs for 1 ns at 300 K.

Figure 3 reports the probability of forming a certain number of NPs as a function of the initial $D_{seed}$ separation and sintering geometry. The final number of distinct MNPs varies, with simultaneous sintering of all four seeds occurring only under certain conditions. Sintering geometry plays a key role—more compact arrangements (Square or Tetrahedron) generally lead to a single nanoparticle of, up to, a relatively large $D_{seed}$. In contrast, for the Line geometry, this happens only when $D_{seed}$ is less than roughly 16 Å; for larger values, the seeds tend to sinter pairwise or in similar, separated ways. This behaviour arises from stochastic, thermostat-induced displacements, which can push seeds beyond the potential’s interaction range. These kicks, together with the different relative orientations of the seeds, are also responsible for the variability of results at fixed geometry and $D_{seed}$.

**Figure 4:**
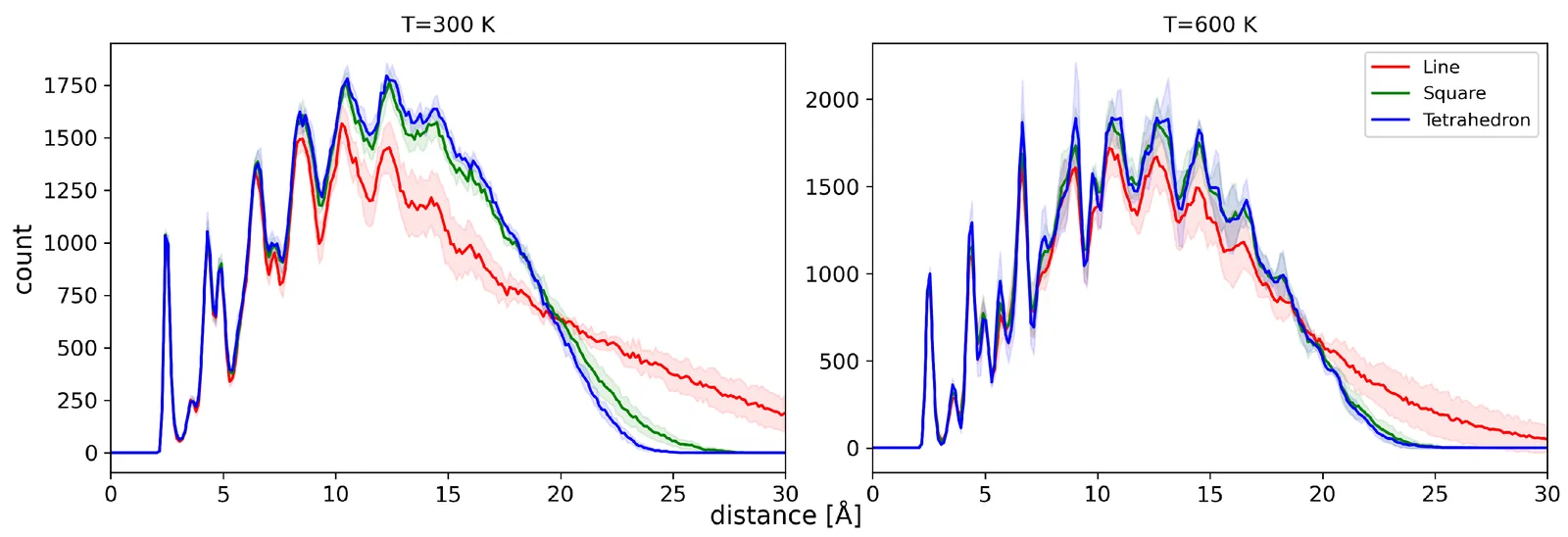
PDDFs profiles for the MNP architectures obtained after 10 ns of simulation with different sintering geometries at 300 K (**left**) and 600 K (**right**). Solid lines and shaded areas represent the averages and standard deviations across multiple runs, respectively. The red and green lines represent the Line and Square geometries, respectively, while Tetrahedron sintering is in blue.

**Figure A5:**
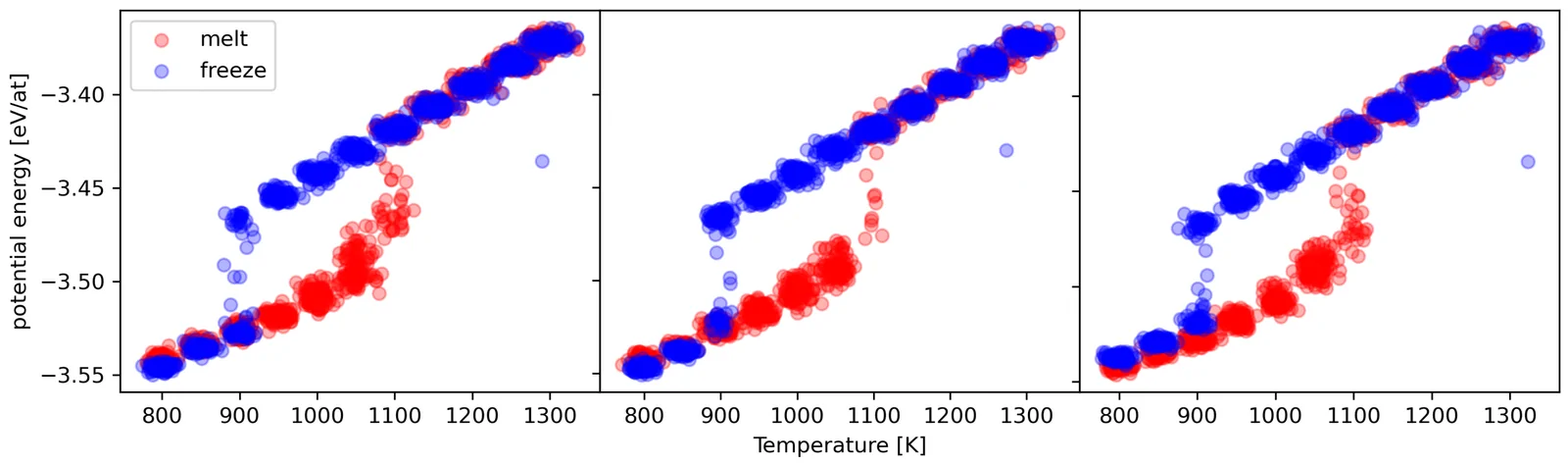
Melting–freezing hysteresis curves for a 588-atoms Cu nanoparticle. The estimated melting temperature $T_{melt}\approx$ 1000 K is well below the sintering temperatures considered in the current work.

**Figure A6:**
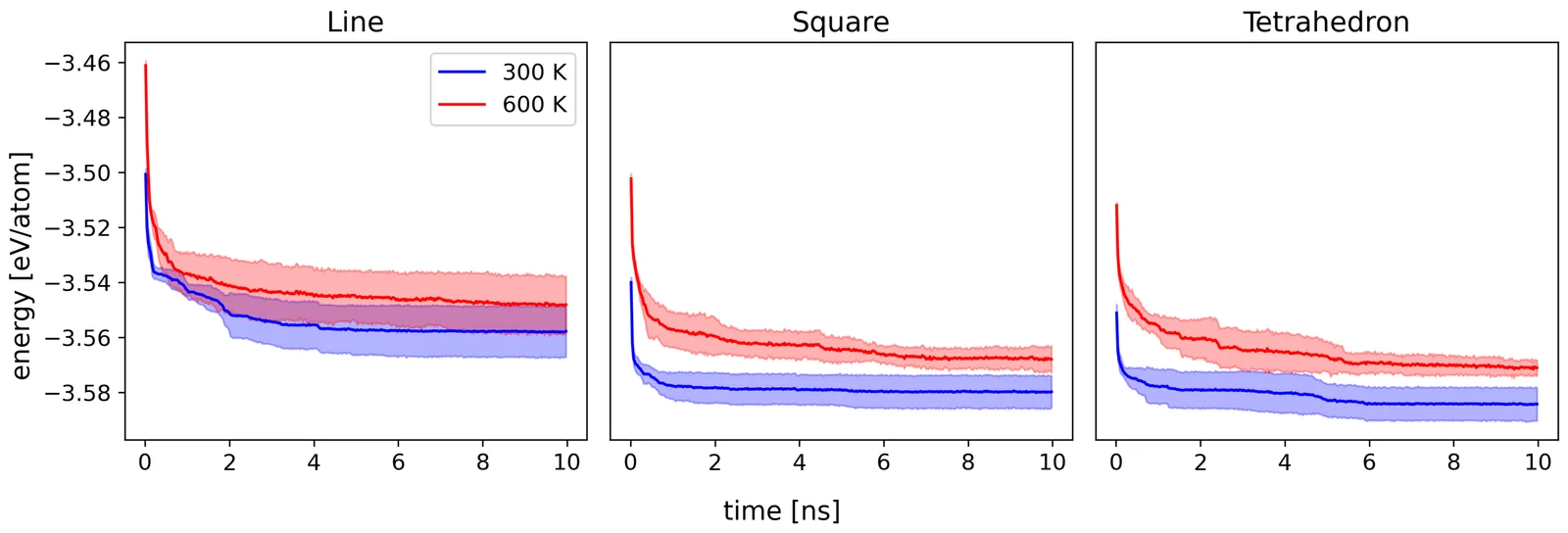
Potential energy per atom in sintering simulations for different initial geometries and thermostat temperatures.

**Figure A7:**
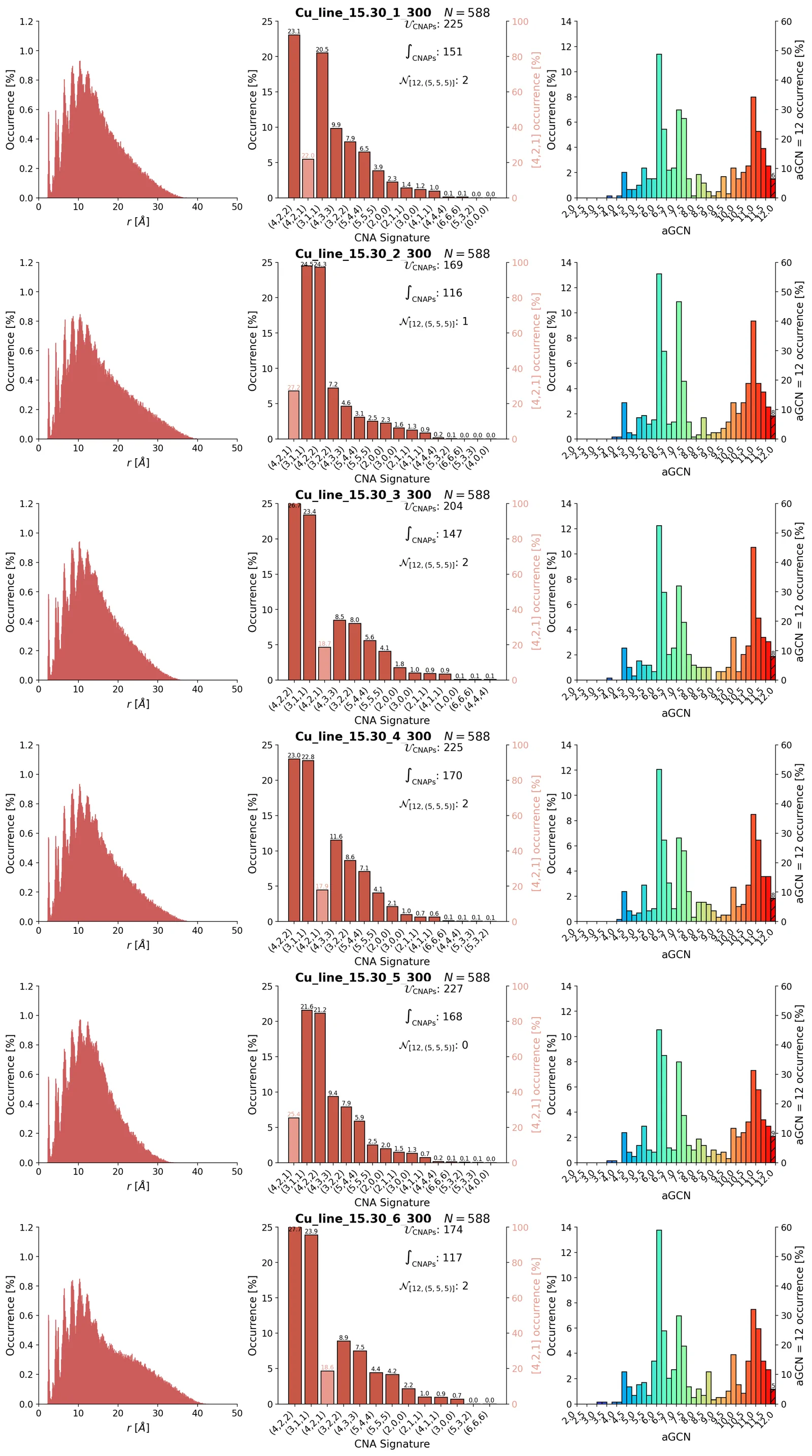
IDs of final structures from simulations with the Line geometry at T = 300 K. Colour-coding as in Figure 2.

**Figure A8:**
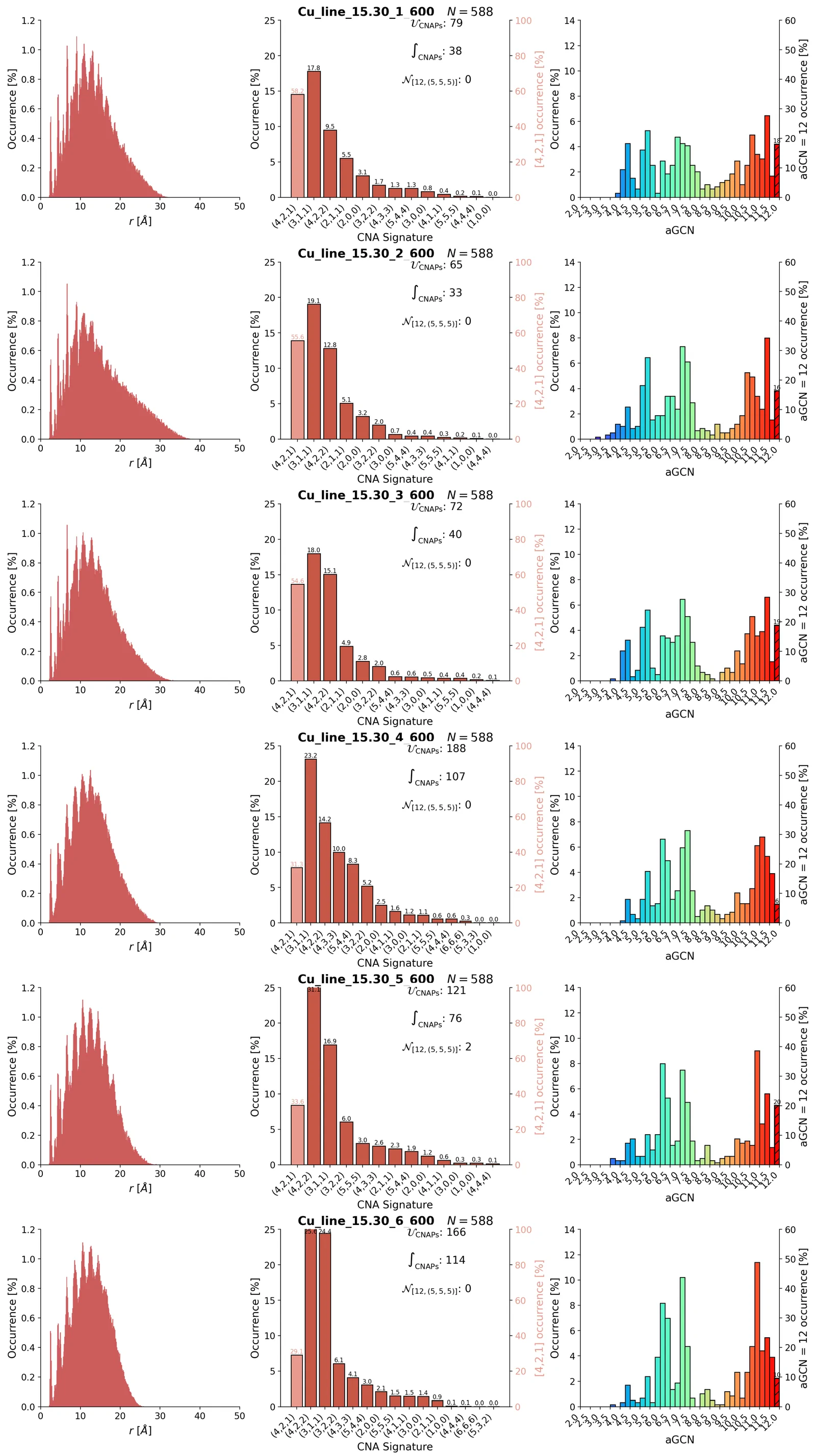
IDs of final structures from simulations with the Line geometry at T = 600 K. Colour-coding as in Figure 2.

**Figure A9:**
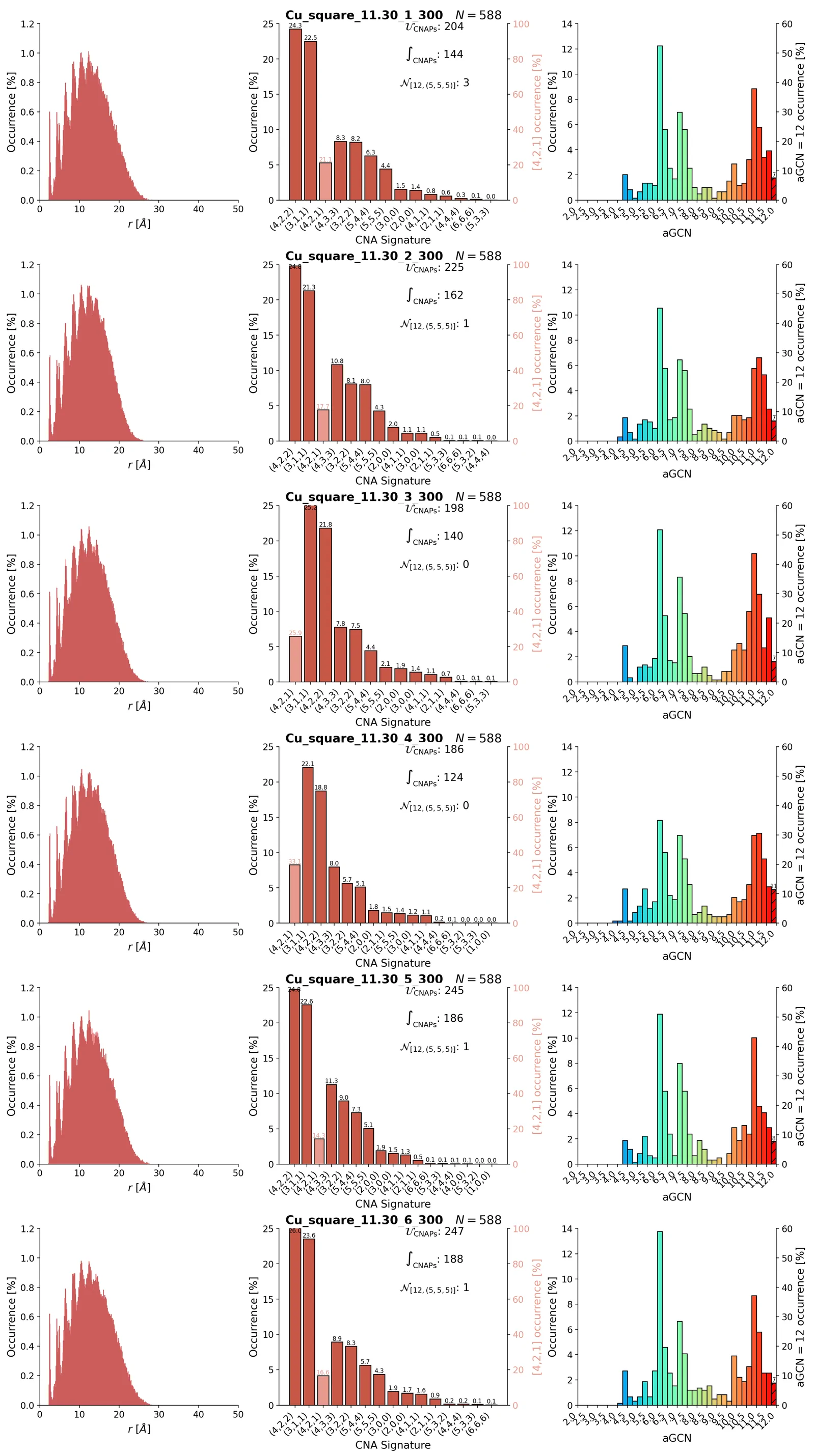
IDs of final structures from simulations with the Square geometry at T = 300 K. Colour-coding as in Figure 2.

**Figure A10:**
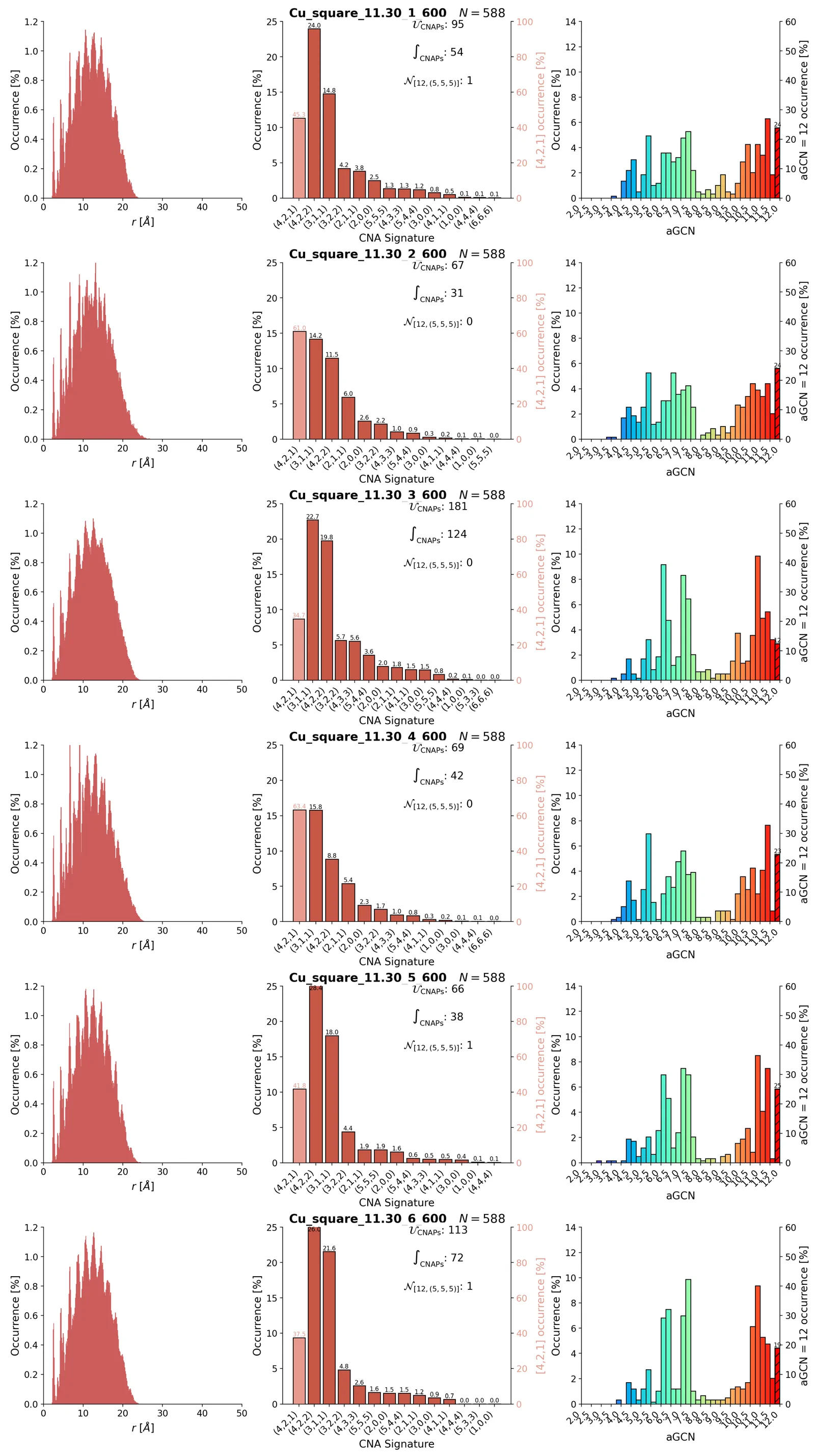
IDs of final structures from simulations with the Square geometry at T = 600 K. Colour-coding as in Figure 2.

**Figure A11:**
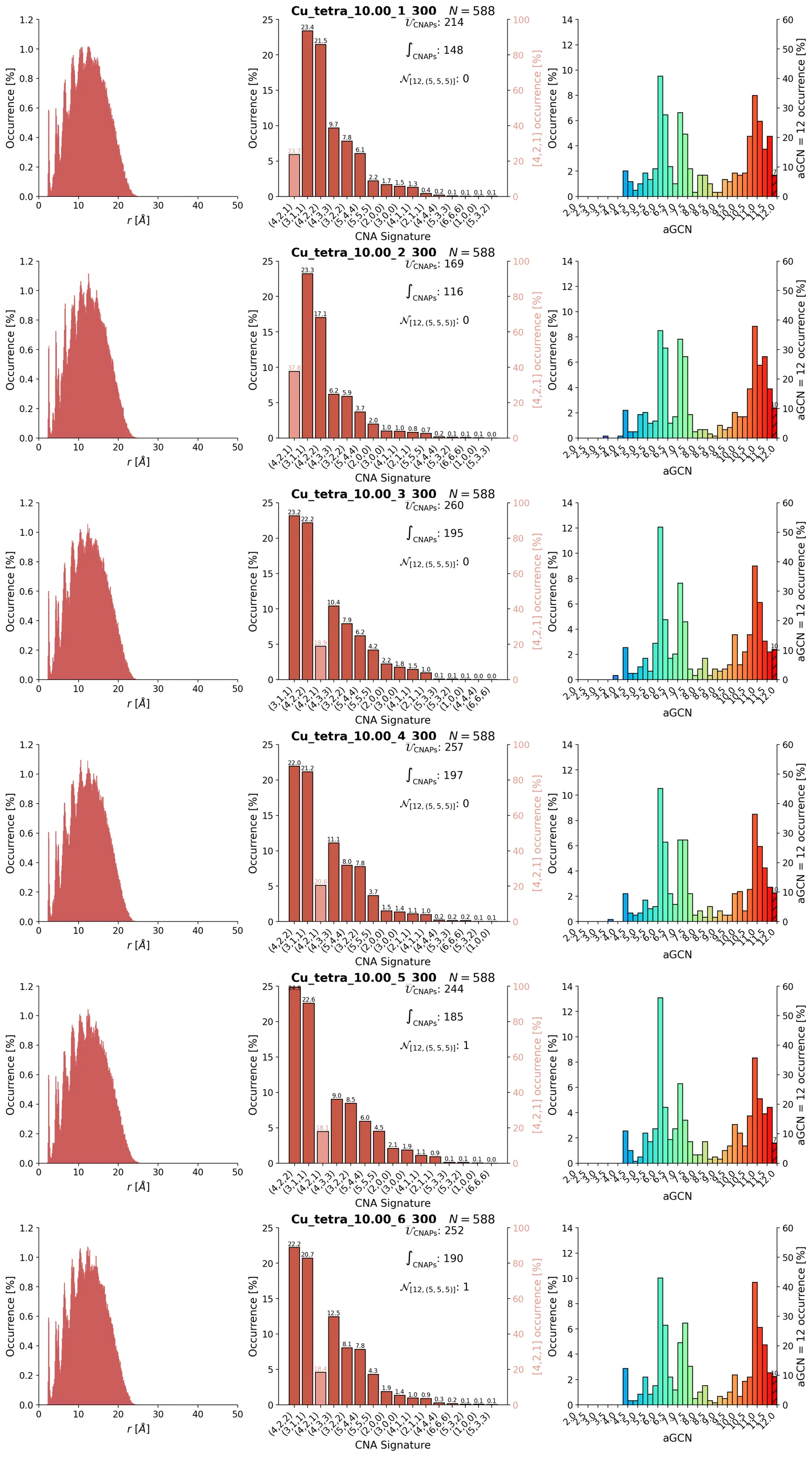
IDs of final structures from simulations with the Tetrahedron geometry at T = 300 K. Colour-coding as in Figure 2.

**Figure A12:**
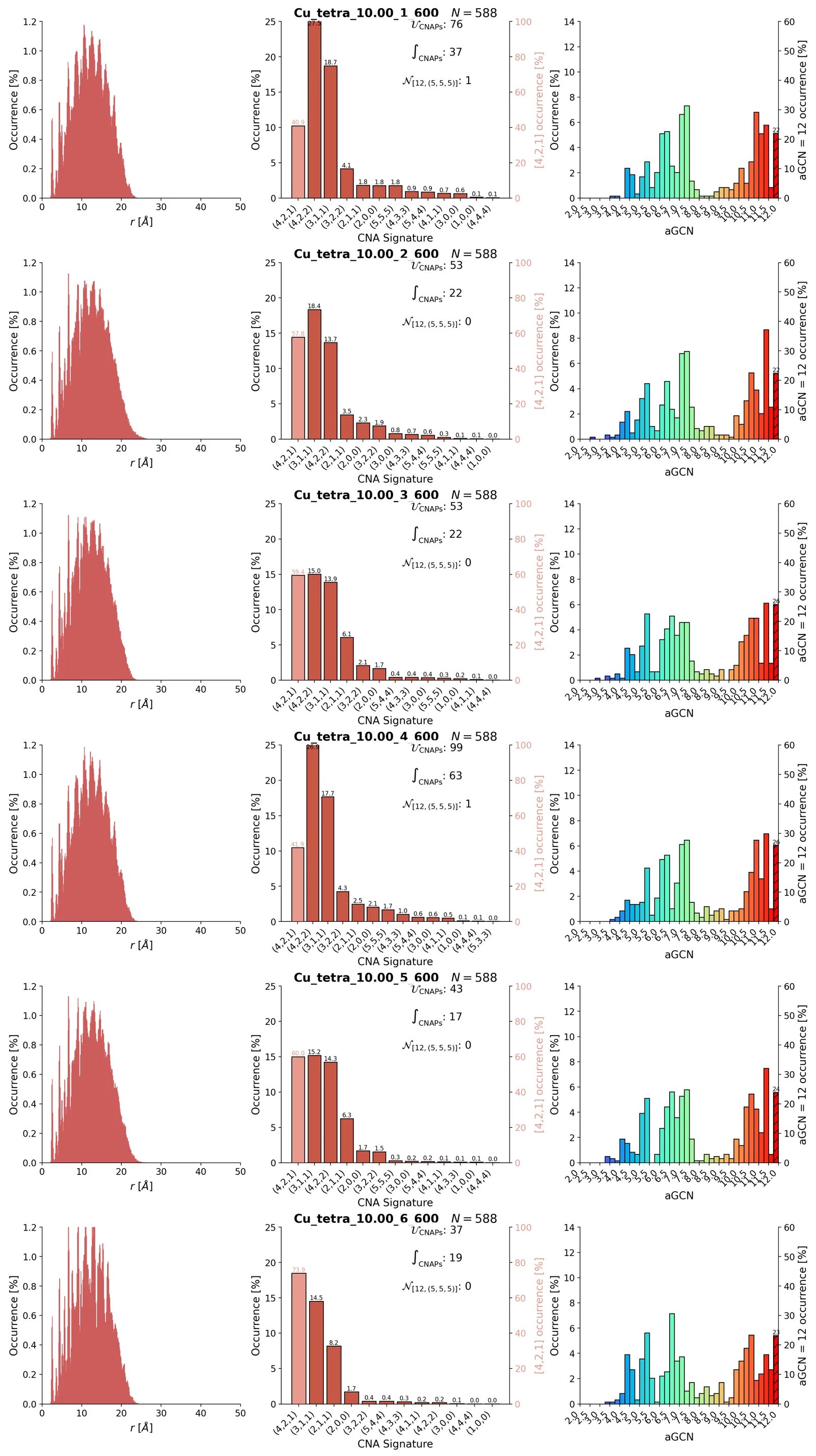
IDs of final structures from simulations with the Tetrahedron geometry at T = 600 K. Colour-coding as in Figure 2.

Then, we performed longer simulations to investigate the dynamics of the sintering processes at 300 K and 600 K. Both temperatures are well below the melting temperature of a 588-atom Cu-NP, as shown from the melting and freezing cycles reported in Figure A5. For each sintering geometry, we ran six independent simulations, ensuring significant variation in the randomised initial relative orientations of the seeds to improve the statistical significance of the results. Energy differences between these isomers are significant, e.g., ≈20 meV/atom (≈11.76 eV in total) when comparing line vs tetrahedron geometry at 300 K (see Figure A6). Let us discuss the architectures obtained from the sintering process and their dependence on the initial geometry of the four seeds. The results of the individual simulations are in the IDs reported in Figure A7, Figure A8, Figure A9, Figure A10, Figure A11 and Figure A12. Looking at the time evolution of the PDDF, we note that after the initial sintering stages, a clear peak corresponding to the lattice parameter suggests the formation of ordered architectures. We note that only one simulation ended in a structure that should be identified as amorphous (inline attachment at 600 K, Figure A8, fourth panel). Figure 4 compares the PDDF of the last snapshots, collected at 10 ns, and averaged over the six simulations per each case.

The impact of the initial sintering geometry is clear, especially on the nanoparticles’ size. At 300 K, the maximum of the PDDF ranges from 25 Å to more than 35 Å, with sizes longer than 32 Å only occurring in the Line case. At 600 K, surface diffusion is faster and tends to smooth differences among the sintering geometries, but it is still not enough, on the 10 ns timescale, to eliminate them. Indeed, we see well-defined peaks up to distance∼18 Å and no size differences between Tetrahedron and Square sintering geometries, with an average MNP size of around 25 Å. In contrast, Line sintering yields sizes of about 30 Å. The PDDF profile at short distances is similar in all cases, with the second peak being more pronounced at 600 K than at 300 K. The long-distance region, distance>6Å, of the PDDF highlights significant structural differences in the MNP shape. While the PDDF drops sharply for the Tetrahedron and Square sintering geometries, it decays smoothly for the Line case (Figure 4).

**Figure 5:**
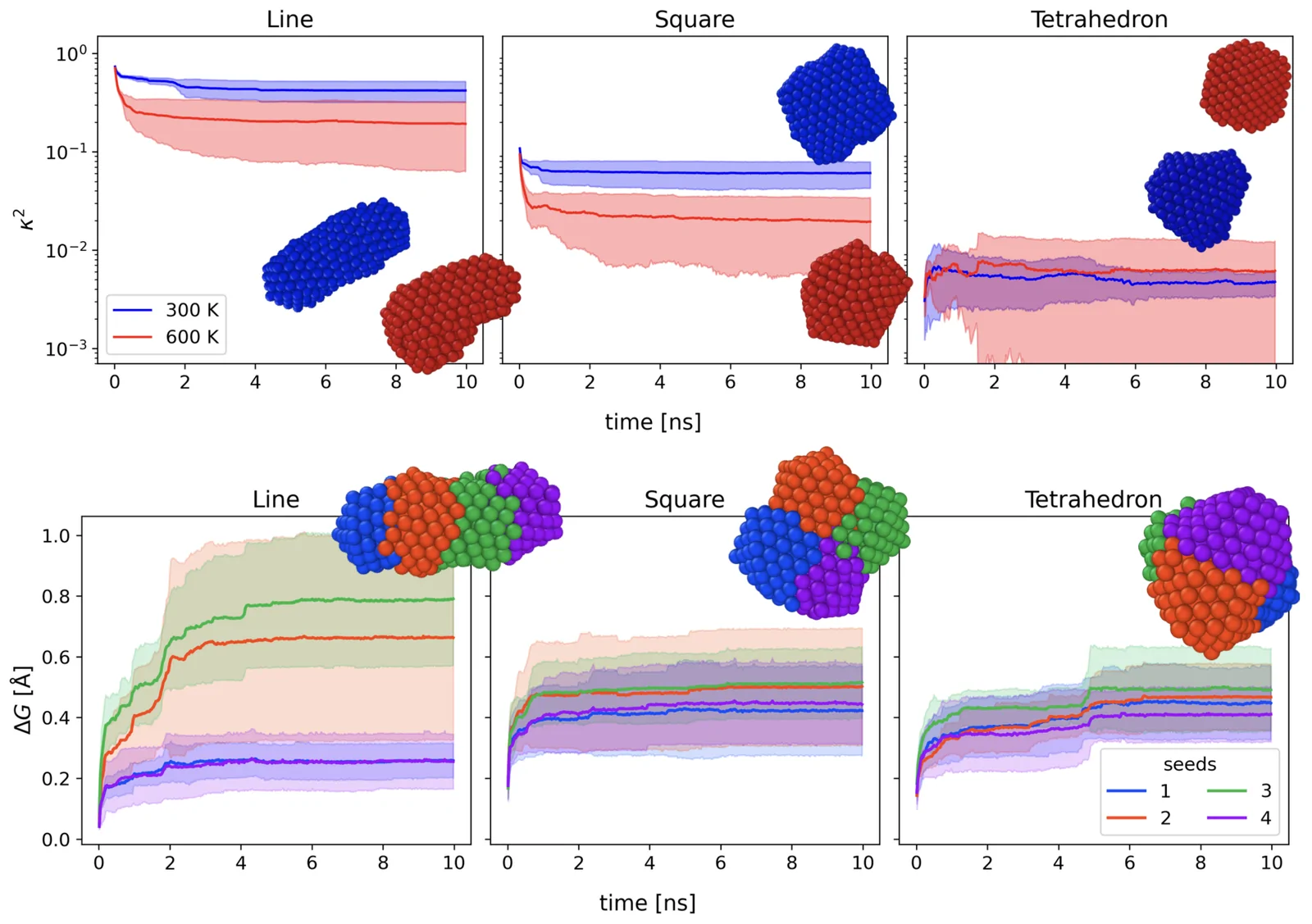
*Top row*: relative shape anisotropy $\kappa^{2}$ as a function of simulation time for different sintering geometries at 300 K (blue) and 600K (red). *Bottom row*: per-seed gyration radius variation at T = 300 K. Final simulation snapshots with per-seed colour-coded atoms to highlight the merging and atoms diffusion among the seeds.

Figure 5 shows the time evolution of key shape descriptors of the grown nanoparticles in terms of $\kappa^{2}$ and the per-seed gyration radius variation, ΔG.

**Figure 6:**
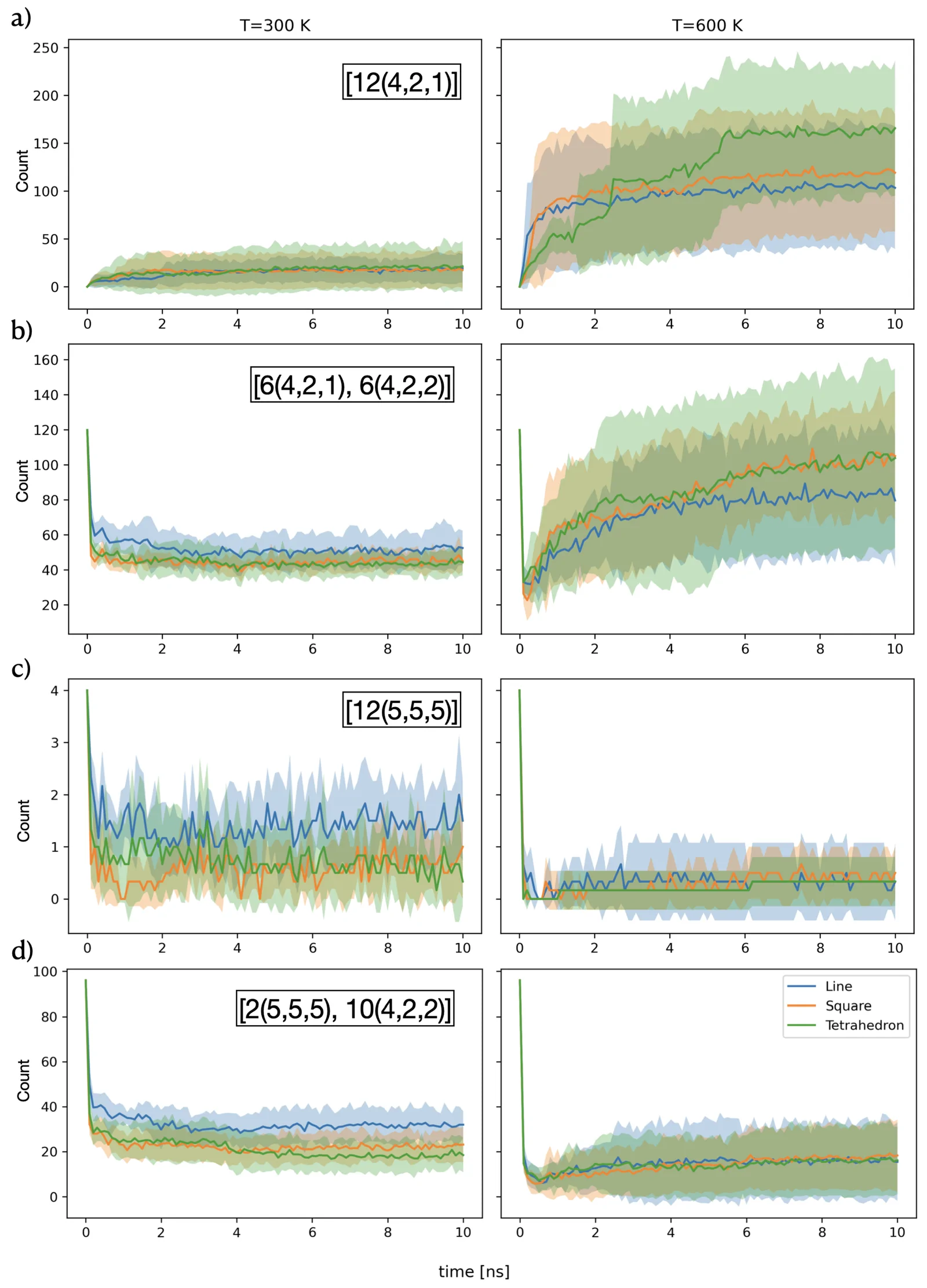
Time evolution of the number of specific CNA patterns at different temperatures and for different sintering geometries. (**a**) [12(4,2,1)] signatures, corresponding to bulk fcc, (**b**) [6(4,2,1), 6x(4,2,2)] signatures corresponding to twinning plane environments, (**c**) [12(5,5,5)] signatures, corresponding to Ih-centre environments, and (**d**) [2(5,5,5), 10(4,2,2)] signatures, corresponding to fivefold symmetry axes environments. Full lines and shaded regions represent the averages and standard deviations across simulations, respectively.

A clear effect of the sintering geometry on the overall shape is evident. Sintering from a Tetrahedron geometry yields spherical nanoparticles ($\kappa^{2}<0.01$) at both temperatures. In contrast, MNPs adopt a prolate shape with $\kappa^{2}>0.1$ when sintered in the Line arrangement. Square-like initial geometries give an intermediate result with a strong temperature dependence. The dynamical mechanisms governing the sintering are also different, depending on the initial sintering geometry, as quantified by ΔG (Figure 5). While sintering from Square and Tetrahedron geometries leads to distortion relative to the initial positions, that is uniform across the seeds, but this is not the case with the Line geometry, where the two innermost seeds change considerably, with the two outermost being significantly less deformed. This analysis highlights how, in this case, the external seeds compress the internal ones, which are deformed by this process, while the former substantially retain their initial shape, as is further highlighted by the number of conserved Ih centres—panel c of Figure 6. We also note the change in ΔG at 5 ns for the Tetrahedron geometry, which reveals a thermally activated process for all MNPs. This change should be related to Figure 6, and indicates an increment in the FCC signature associated to a restructuring of the MNP.

Figure 6 helps in terms of quantifying structural ordering and local atomic environments. The individual CNA and aGCN analysis for each individual simulation is in the middle and right columns of Figure A7, Figure A8, Figure A9, Figure A10, Figure A11 and Figure A12. As expected, structural rearrangements depend on temperature, but they also differ due to the sintering geometry. At 300 K, the contribution of FCC-bulk atoms is lower than in other cases. The counts of CNA patterns for FCC, twinning plane, and fivefold-axis environments remain stable after a brief initial variation, and the number of conserved Ih centre environments is not cancelled, indicating that the initial seed structure is largely unchanged. As reported in Table A2, if the sintering occurs simultaneously on different facets of the seed(s), it leads to structures with a higher degree of disorder, as quantified by the number of $N_{CNAP}$ and $U_{CNAP}$, with a clear trend as Tetrahedron > Square > Line at 300 K. Furthermore, almost 40% of the atoms in MNP sintered from a Tetrahedron geometry have their own local environment, and among them, three quarters are defects. At 600 K, however, there is sufficient kinetic energy to allow restructuring, as several diffusion mechanisms are activated. In this case, the number of different patterns is much lower, but still clearly depends on the initial geometry (Line > Square > Tetrahedron). Prominent Ih-related patterns rapidly drop (right column of Figure 6), with [12(5,5,5)] being zero below 1 ns, showing that the initial atomic environment-level structure is lost. FCC and twinning-plane environments grow significantly throughout the simulations as internal ordering develops. The kinetics differ between the sintering geometries. Line and Square show similar timescales for forming the first FCC-like environments, but are less prone to increasing them. The combination of a high final count of twinning-plane environments with the near-complete absence of Ih centres and the presence of fivefold axes suggests the possible formation of decahedral structures as an outcome of these simulations, as is, in fact, observed (e.g., in Table 1).

We can identify some trends based on the initial sintering geometry. The more compact this is (i.e., Tetrahedron), the larger the contact area. A tetrahedral sintering geometry starts with twelve misaligned (111) facets being in contact, compared with the six in a Line geometry. In other words, the shared area among seeds for a Tetrahedron is about twice that for a Line. The first activated mechanism is likely adatom surface diffusion on (111), which has a low activation energy barrier. More anisotropic sintering geometries (i.e., Line) sphericise and rearrange slowly because adatoms and vacancies should travel longer distances. At low temperatures, they yield higher structural ordering because they preserve their original seed morphology. A large contact area increases interfacial strain. At high temperatures, the seeds are more likely to undergo complete structural rearrangements and rapidly evolve into a unique, highly spherical shape.

## 4. Conclusions

We present a new and open source software package, NPGrowth, to model seed-mediated growth on metallic nanoparticles in various situations, including those that can lead to dendritic, spiky, chiral, and non-spherical nanoparticles. The key feature of NPGrowth is a modification of LAMMPS’ fix deposit that allows simulation of a variety of deposition routes compatible with LAMMPS features, including machine-learning potentials.

We aim to introduce the capabilities of the proposed package, not to exhaust or investigate all cases but to show the effect of directionality and the geometry of atomic/seeds sources for the synthesis of copper nanoparticles.

We discuss atomic deposition with well-defined spatial patterns onto cubic and truncated octahedral seeds, showing how to set up NPGrowth source and target regions to get both branched, star, concave and flattened/oblate motifs. We propose a morphological classification of the obtained nanoparticles employing the pySNOW descriptors. We introduce a nanoparticle ID that includes the PDDF, the CNA and the aGCN distributions plus a few shape and structural quantities. We stress that shape and structure here refer to the envelope enclosing the nanoparticle and to its geometrical shape, respectively. The nanoparticle geometrical character, instead, refers to whether it belongs to FCC, FCC twin, or multiple-twinned (Ih, polyIh, and Dh) geometry. Our examples show the variety of morphologies we can generate with NPGrowth, but they are not exhaustive.

We then analyse in detail the sintering of four Cu-Ih nanoparticles, each with 147 atoms, soft-landed onto one another in three different orientations or sintering geometries. We show an effect on the size distribution due to orientational attachment, with a higher probability of forming a single nanoparticle for 2D or 3D sintering geometries. Inline attachment favours two-by-two aggregation of seeds. In soft-landing sintering at short relative distances among seeds, the initial geometry affects the final shape of the nanoparticle. Analysing the first 10 ns after sintering, we show that inline attachment leads to strongly elongated shapes, even at 600 K. A planar attachment, modelled as squarely displaced seeds, generates highly faceted but concave shapes. Three-dimensional, isotropic (Tetrahedron) soft-landing favours the formation of an individual, mostly spherical, convex, architecture. Significant internal restructuring occurs depending on the system temperature, and a detailed analysis of the CNA signatures and patterns allows a deep understanding of the underlying physical processes. This analysis shows that the kinetic timescale depends on sintering geometry, with a 5 ns window for major FCC reordering in 3D Tetrahedron sintering while the kinetics is slower for the other initial geometry.

We believe NPGrowth will help those in the scientific community working on nanosystems to gain atomistic insights into their formation processes and build a sufficiently diverse structural database to screen the best candidates from a large set. Furthermore, the proposed pySNOW characterisation and classification should establish a common nomenclature for identifying nanoparticle morphology (size, shape, and motif).

## Figures and Tables

**Table 1:** NPGrowth examples and relative settings, definition of the initial seed, and following pySNOW characterization. The last column reports the name given to the easily identified final structures. The ∗ highlights cases where different names are assigned although similar shape descriptors because the CNA and aGCN distributions differ; see Figure 2.

NPGrowth Set-Up	Initial Seed	*N*	*f*	$\kappa^{2}$	α	Shape Identification	Structural Taxonomy	Name
1-by-1 random from 100 Å seed-centred cubic SR; CoM-target; ρ=0.5THz	Cb_4361_	9761	3	$4\times 10^{-5}$	0.6	Compact-spheroid	FCC	Concave Cb
6-by-6 from 6 fixed SRs at 310 Å and normal to the initial cube facets; CoM-target; ρ=0.5THz	Cb_4361_	9761	1.6	$1.1\times 10^{-2}$	0.42	Branched	FCC	Spike
8-by-8 from 8 fixed SRs at 40 Å from CoM, normal to (111) facets; CoM-target; ρ=0.005THz	TO_201_	465	2.5	$2.1\times 10^{-2}$	0.64	Compact-oblate	FCC	Onigiri ∗
5-by-5 from 5 fixed SRs lying on a xy plane at 40 Å from CoM; CoM-target; ρ=0.42THz	TO_586_	856	2.6	$3.1\times 10^{-2}$	0.57	Compact-oblate	FCC	Star ∗
Sintering line geometry, T = 300 K	four Ih_147_	588	2.3	$5.0\times 10^{-1}$	0.58	Compact-prolate	Poly-Ih	Elongated
Sintering tetrahedron geometry, T = 600 K	four Ih_147_	588	2.83	$3\times 10^{-3}$	0.70	Compact-spheroid	Dh	Dh

**Figure A1:**
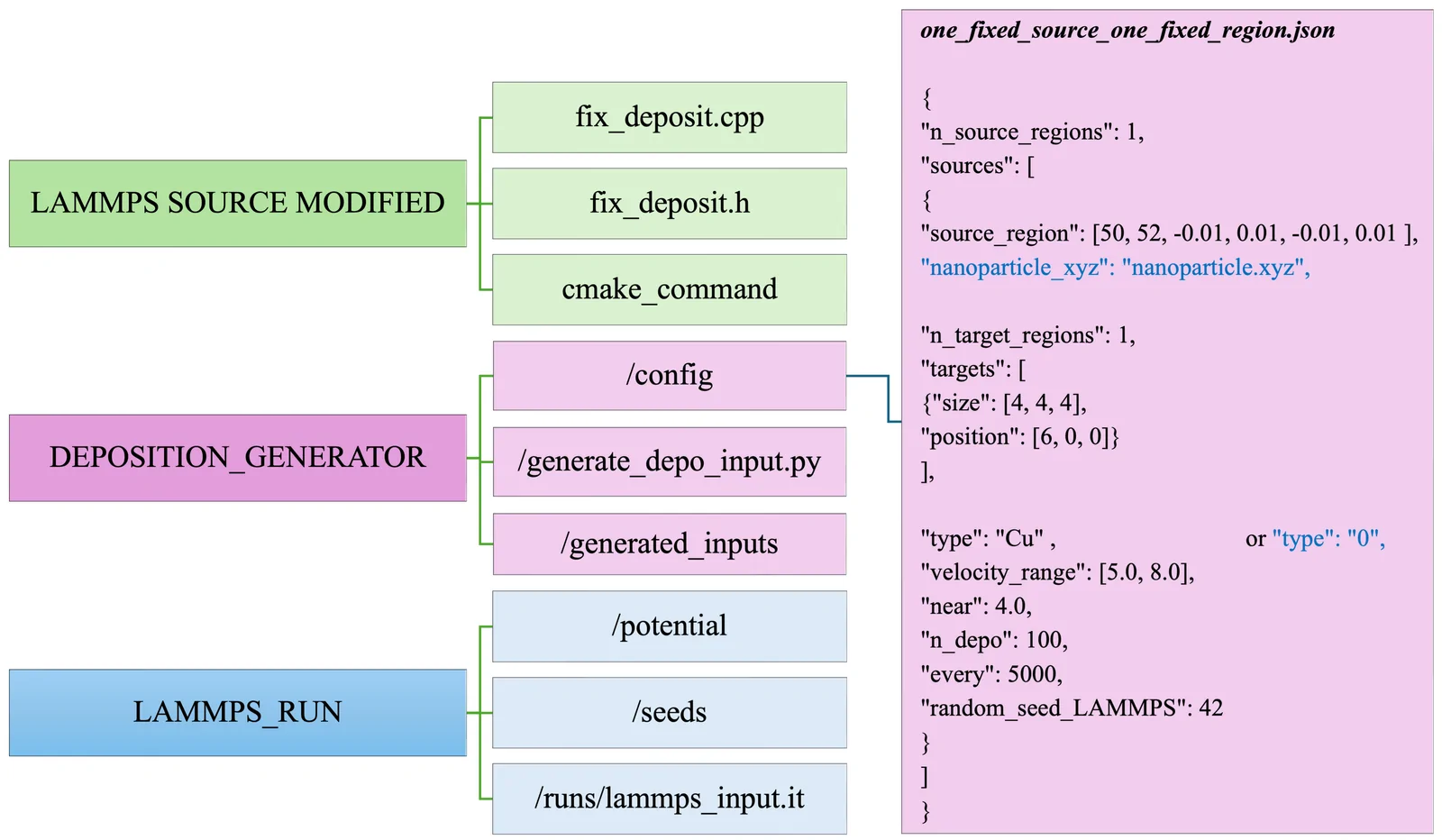
NPGrowth workflow and an example JSON configuration file for the simplest deposition setup, consisting of one fixed source and one fixed target. The example illustrates the input parameters required to configure NPGrowth, which are described in detail in the text.

**Figure A2:**
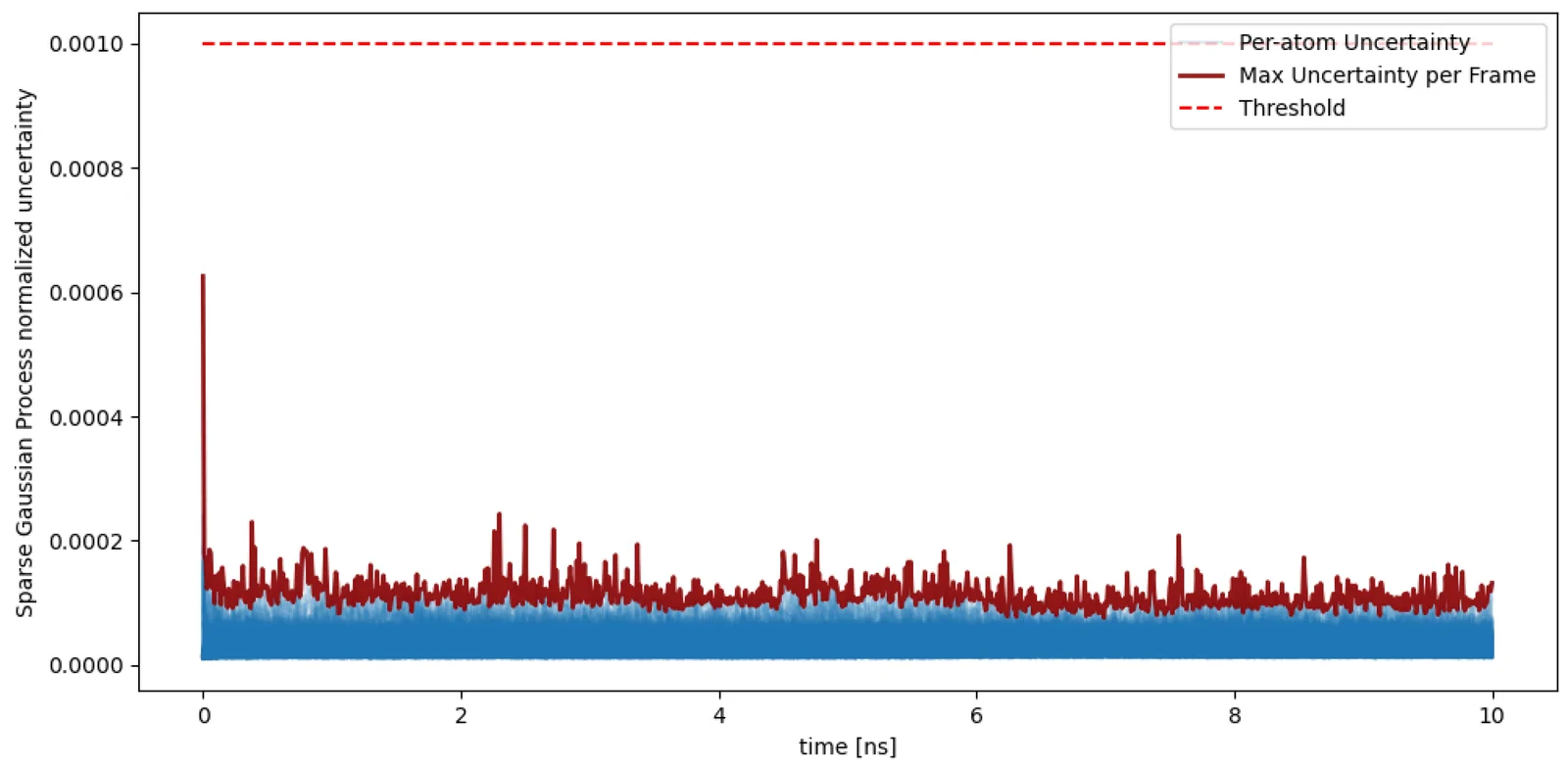
Sparse Gaussian process normalized uncertainty as a function of simulation time in an example 1 ns line sintering simulation: faint blue lines show the uncertainty of each atom, and the solid dark line shows the maximum uncertainty in each frame. The red dashed line highlights the threshold to label configurations as uncertain and add them to the training set: during this simulation uncertainty values remain well below threshold.

**Figure A3:**
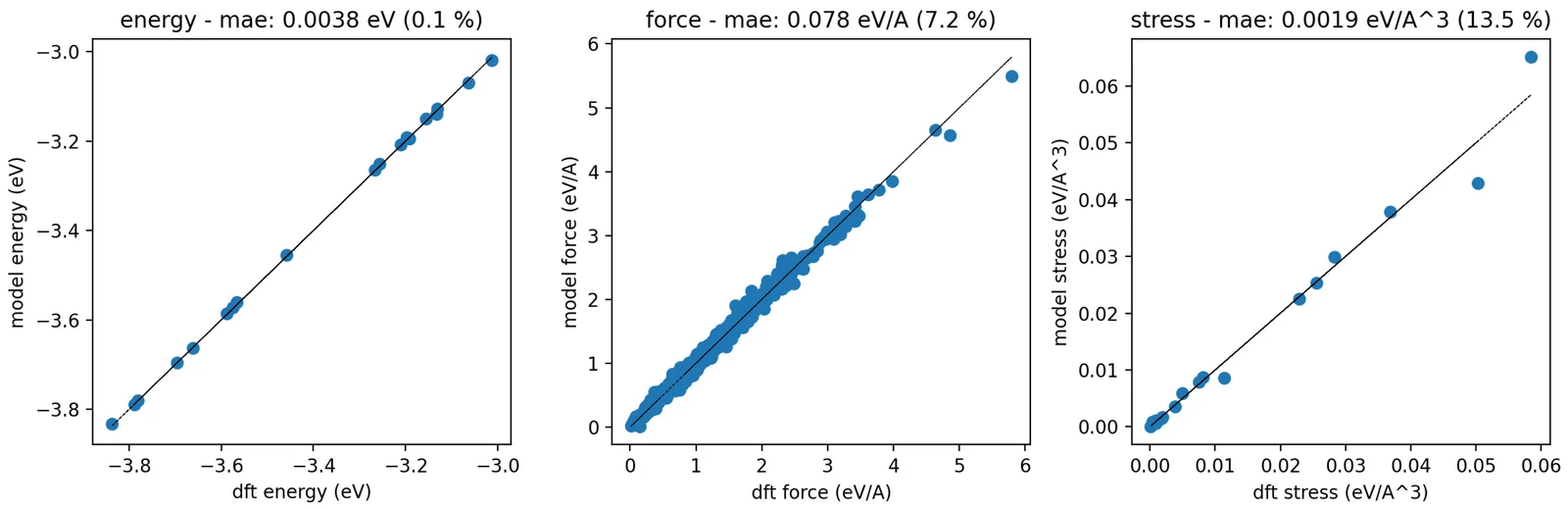
Parity plots of test-set configurations for energy, forces, and stress with the employed potential. In the title of each plot are reported the mean absolute error (mae) and, in parentheses, the mean absolute error/mean absolute value ratio.

**Figure A4:**
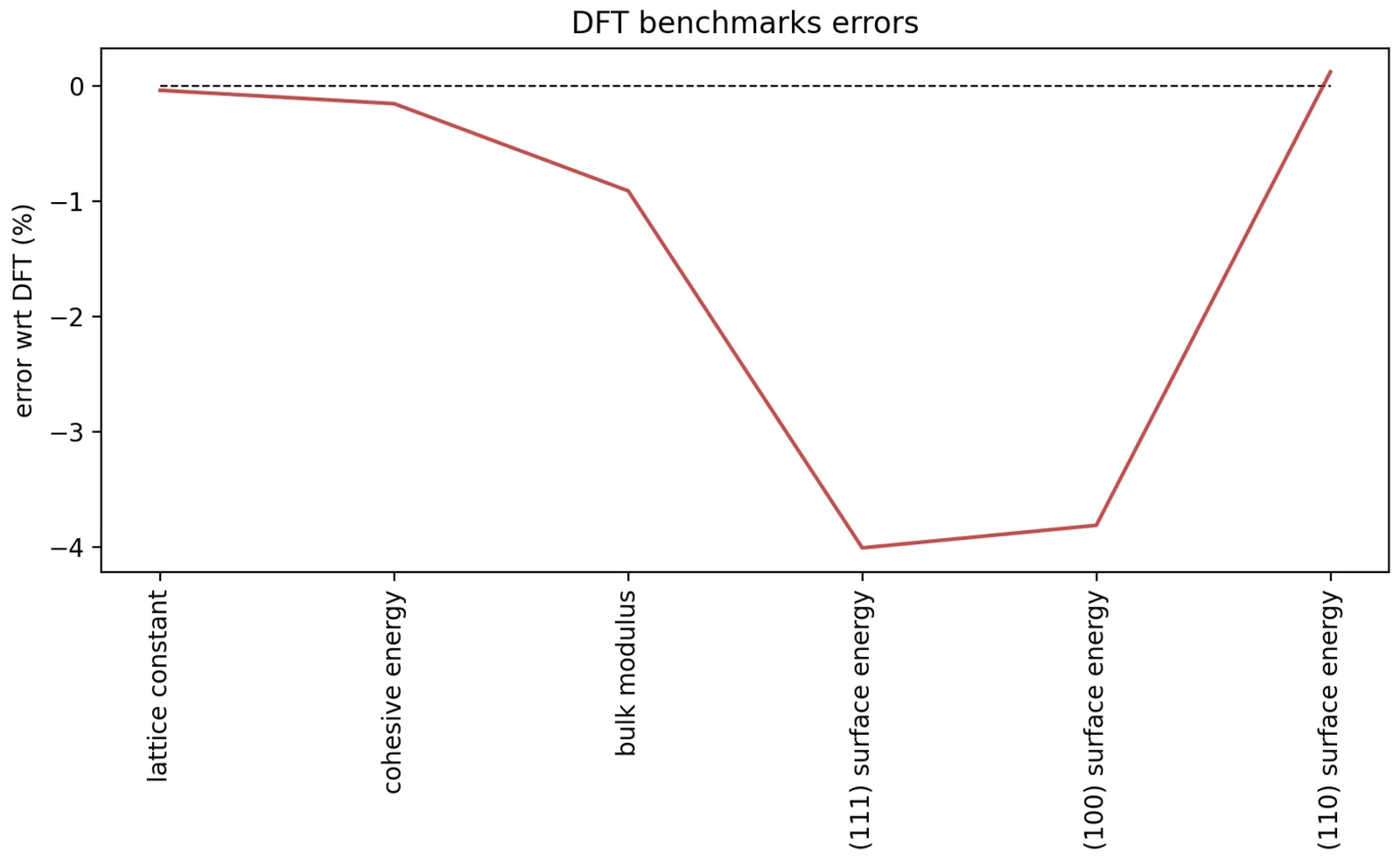
Relative percentage error of the MLP in reproducing key quantities computed at the reference DFT level.

**Figure A13:**
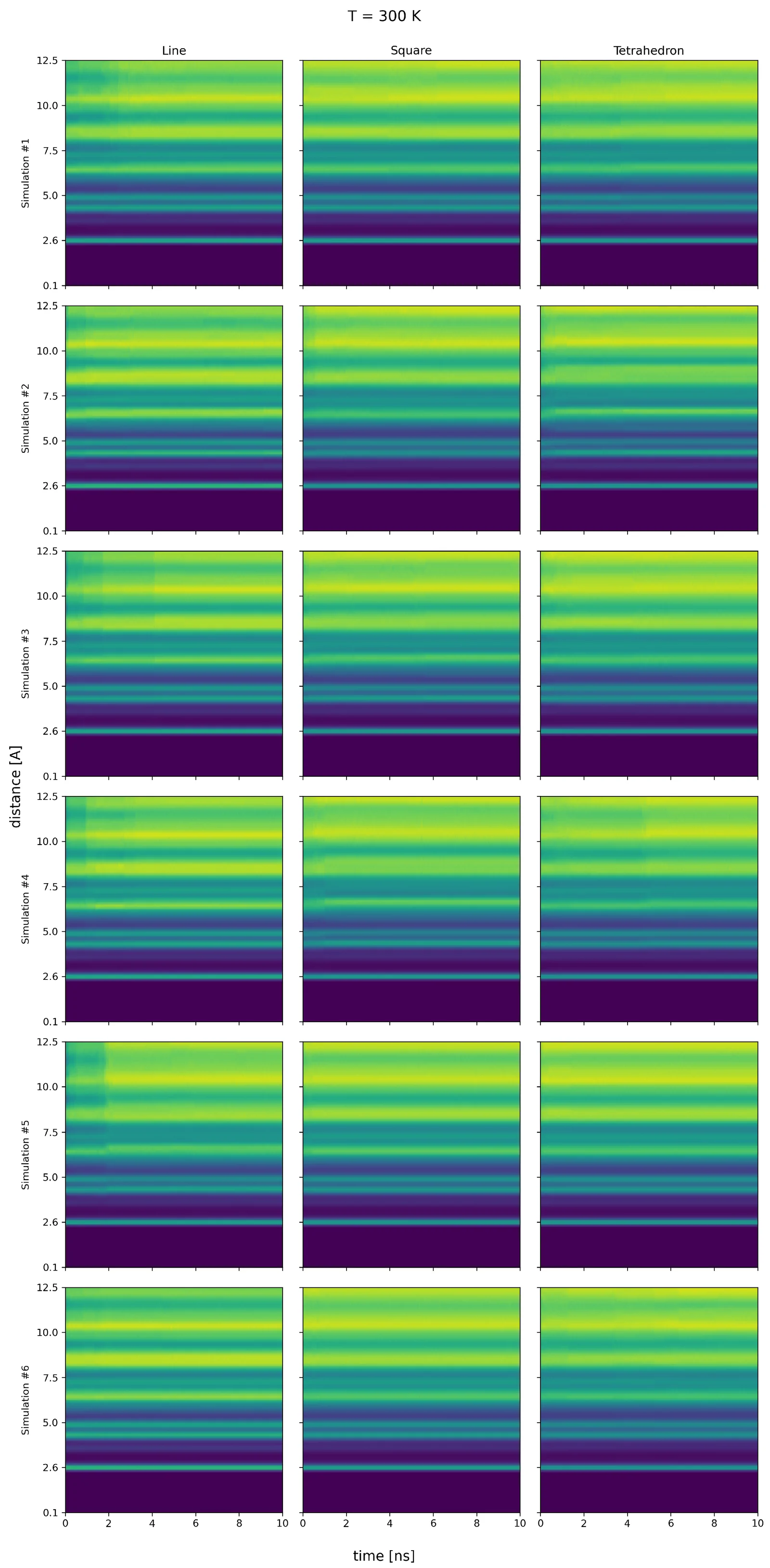
PDDF time evolution during sintering simulations at T = 300 K. Colour refers to occurrence of pairs at a certain distance, from blue (no occurrence) to yellow (max value = 2000). We note that at low T, the PDDF profile are quite similar, with mild deviations at 5 Å.

**Figure A14:**
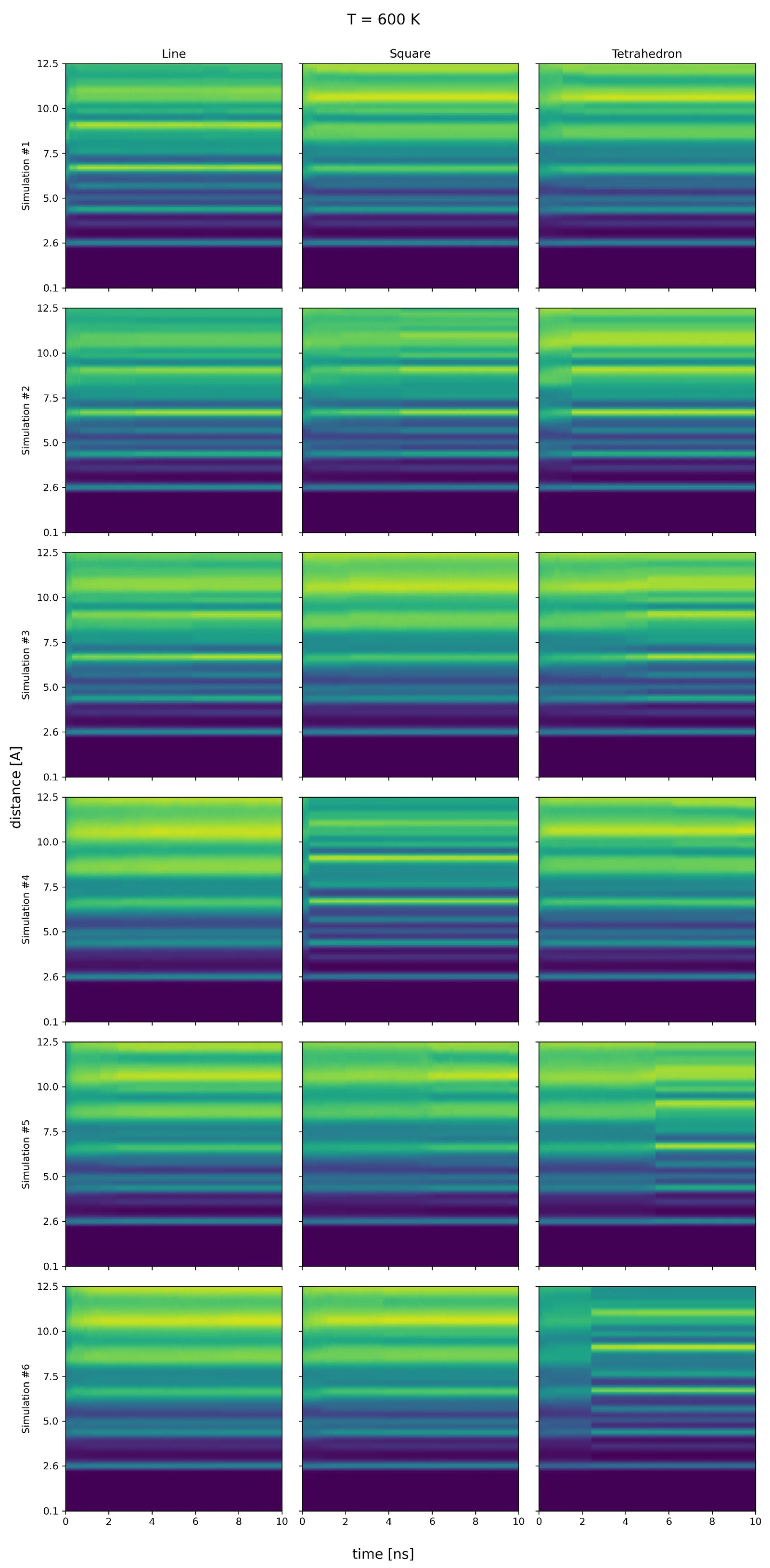
PDDF Time evolution for the sintering simulations at T = 600 K. Colours as in Figure A13. The PDDF differ for all the cases, supporting the presence of various structural motifs, as discussed.

## Data Availability

Our codes are available at https://github.com/nanoMLMS (accessed on 22 September 2026). At the page https://github.com/nanoMLMS/NPGrowth_V2 (accessed on 22 September 2026) the interest Reader could find the input file used for the toy model. Cu-sintering trajectories presented in the study are openly available in Dataverse Unimi, at https://doi.org/10.13130/RD_UNIMI/2D3PW9.

## Abbreviations

The following abbreviations are used in this manuscript:

MNP	Metallic Nanoparticle
cMD	Classical Molecular Dynamics
MLP	Machine Learning Potential
EAM	Embedded-Atom Method
RGL	Rosato–Guillope–Legrande
SR	Source region
CNA	Common Neighbour Analysis
CNAP	Common Neighbour Analysis Pattern
aGCN	Atop Generalized Coordination Number
PDDF	Pair Distance Distribution Function
RAD	Radial Atomic Density
CoM	Centre of Mass
FCC	Face Centred Cubic
Ih	Icosahedron
Dh	Decahedron

## Appendix A. NPGrowth Overview and Inputs

The NPGrowth_V2 workflow is designed to guide the user through the preparation and execution of nanoparticle growth simulations, from the installation of the required software to the generation of the deposition input file and the subsequent simulation run. The main steps of this procedure are summarized in Figure A1. The code is openly available on GitHub at https://github.com/nanoMLMS/NPGrowth_V2 (accessed on 22 September 2026) with more input examples and tutorials.

After cloning the repository and completing the installation using the modified LAMMPS source files, the user can access the core component of NPGrowth_V2: the DEPOSITION_GENERATOR directory; while the LAMMPS_RUN directory contains the files required to perform the simulations with LAMMPS, including (i) the simulation input file, specified in LAMMPS format at runs/lammps_input.in, (ii) the initial seed onto which deposition takes place, and (iii) the interatomic potential.

The deposition process is configured through the DEPOSITION_GENERATOR procedure. The user first prepares a JSON file containing the required deposition parameters. This JSON file is then provided as input to generate_depo_input.py, together with the destination path for the generated file. The script produces a LAMMPS-formatted input file containing the instructions required to perform the deposition simulation. The parameters required in the JSON configuration file are described below. An example of atomic one-by-one deposition from a fixed source to a fixed target is shown in Figure A1.

- “n_source_regions”: specifies the number of source regions to be defined by the user.
- “sources”: defines the properties of each source region. When multiple sources are used, they are specified as an array of JSON objects, with each object enclosed in curly braces and separated by commas.
  The first parameter, “source_region”, defines the spatial extent of the source as $\left[x_{1},x_{2},y_{1},y_{2},z_{1},z_{2}\right]$. These coordinates specify the lower and upper bounds along each Cartesian direction, thereby determining the position and dimensions of the source region. “nanoparticle_xyz” is an optional parameter used for sintering simulations. When specified, it provides the path to the XYZ file containing the atomic coordinates of the nanoparticle to be deposited.
  Each source must also be associated with a target region. The target can consist of a single rectangular parallelepiped or a union of multiple parallelepipeds. The n_target_regions define how many target regions are expected. A target is defined as a list of parallelepipeds, and deposition is directed on the lines connecting the centre of the source to the centre of each target region. Each target parallelepiped is specified by its dimensions, “size”: $\left[l_{x},l_{y},l_{z}\right]$, and the coordinates of its centre, “position”: $\left[p_{x},p_{y},p_{z}\right]$.
- The type of deposited object must be specified. For atomic deposition, this parameter identifies the chemical element of the deposited atoms. For sintering simulations, the value must be set to 0.
- “velocity_range”: specifies the range of initial velocities assigned to atoms extracted from the source, expressed in Å/ps.
- “near”: specifies the minimum allowed distance between a newly inserted atom and the atoms already present in the simulation. This parameter prevents the insertion of atoms at excessively short distances, thereby avoiding unphysically large repulsive forces and potential numerical instabilities.
- “n_depo”: specifies the total number of atoms or molecules to be deposited from the corresponding source.
- “every”: specifies the number of integration steps between successive deposition events from a given source. The corresponding physical time interval is obtained by multiplying every by the integration timestep, Δt.
  For one-by-one deposition, the deposition rate is, therefore,
  $$R=\frac{1}{\mathrm{every}\;\Delta t}.$$
  For an *N*-by-*N* deposition scheme, in which $N_{\mathrm{source}}$ objects are deposited per event, the rate becomes
  $$R=\frac{N_{\mathrm{atoms}}}{\mathrm{every}\;\Delta t}.$$
  When expressed in units of objects per integration step, the factor Δt is omitted.
- Finally, the user must provide a random_seed_LAMMPS for the stochastic operations performed by LAMMPS. Using the same seed and simulation parameters enables reproducibility, whereas changing the seed generates a different stochastic realization under the same conditions. Independent realizations can, therefore, be used to accumulate statistics and assess the variability of the simulation results.

Some considerations should be taken into account when defining the deposition geometry. First, the deposition direction is determined by the line connecting the source region to the target region, without explicitly accounting for the geometry of the initial nanoparticle seed. Consequently, if the selected source and target coordinates define a trajectory that intersects the seed, the deposited atoms or nanoparticles will encounter the first surface along that trajectory, which may differ from the intended deposition site. For this reason, when using random source regions, we recommend directing deposition toward the centre of mass of the initial seed. This choice reduces the likelihood of unintended deposition trajectories and helps ensure that the resulting growth process is consistent with the intended simulation setup.

## Appendix B. Machine Learning Potential for Cu Nanoparticles

The machine learning potential (MLP) employed in this work was trained using the FLARE code [62,63], and tested with the mlputils package (https://github.com/gilbogilb/mlp_codes_utils) (accessed on 22 September 2026). The MLP is trained on a custom-built dataset of configurations gathered with on-the-fly MD runs, as explained in ref. [62], the content of which is described in Table A1. The dataset includes configurations from bulk fcc and liquid systems, low-index surfaces, and several nanoparticles spanning different sizes and structural motifs, including both fcc-like and fivefold-coordinated environments, to ensure extensive coverage of atomic environments possibly found during production.

To verify a posteriori this assumption, we monitored the normalized uncertainty of the sparse Gaussian process (Equation 18 of Ref. [63]) throughout a representative 1 ns simulation of the sintering of four seeds arranged in a linear geometry. As shown in Figure A2, the uncertainties during the simulation remain well below the threshold that, during on-the-fly MD, would have classified a configuration as uncertain and triggered its inclusion in the training database.

We have employed the B2 term of Atomic Cluster Expansion [68] as descriptors, with squared normalized dot product as the GP kernel and the following hyperparameters: radial basis size $n_{max}=8$, max angular basis terms $l_{max}=4$, and cutoff radius $r_{cut}=4.9$ Å.

The energy, forces and stress of these reference atomic configurations was computed within the density functional theory framework using PBEsol as the exchange-correlation functional. We used a wavefunction cutoff of 55 Ry, a charge density cutoff of 440 Ry, and employed electronic smearing within the Marzari–Vanderbilt scheme [69] with a smearing parameter of 0.2 eV. All calculations were performed with Quantum Espresso [70,71].

The potential is evaluated by means of its test-set errors and errors on reproducing key derived quantities computed with DFT (cohesive energy, lattice constant, bulk modulus, and surface energy of low-Miller-index surfaces). The results are shown in Figure A3 and Figure A4.

**Table A1:** Dataset composition for training and testing the machine learning potential employed in the sintering simulations.

Configuration Type	Train	Test
Bulk fcc	16	2
Bulk liquid	15	1
111 surfaces	17	1
110 surfaces	11	0
100 surfaces	8	2
liquid surfaces	21	3
55-atoms Ih	6	3
147-atoms Ih with 3 adatoms	4	0
55-atoms Ih + detached atom	27	3
44-atoms Oh	13	3
85-atoms Dh	10	0
110-atoms defected Ih	6	0
55-atoms droplet	12	0
110-atoms droplet	4	1
44-atoms droplet	6	0
63-atoms droplet	8	0
130-atoms droplet	2	0
Total	186	19

## Appendix C. Sintering Simulations

Sintering simulations are performed at T = 300 K and T = 600 K. Both these temperatures are well below the melting temperature of a 588-atom cluster ($T_{melt}\approx 1000$ K). The melting temperature is estimated from melting–freezing hysteresis curves obtained with iterative MD simulations [67]. These are shown in Figure A5.

### Appendix C.1. Potential Energy of Sintered Configurations

We report here the evolution of the per-atom potential energy in sintering simulations. The plot in Figure A6 shows that a significant energy difference is present between sintered nanoparticles starting with a different geometry.

### Appendix C.2. Final Structures Analysis

Table A2 contains statistical informations about the values of $N_{CNAP}$, ∫CNAP and their ratio for the last snapshots of the simulations.

**Table A2:** Number of unique CNA patterns ($N_{CNAP}$), singleton patterns ($U_{CNAP}$) and $U_{CNAP}/N_{CNAP}$ ratio for the final snapshot of sintering simulations. For all represented quantities, the main value is the average across the 6 simulations and the value in parentheses is the standard deviation across simulations.

	T=300 K			T=600 K		
	Line	Square	Tetrahedron	Line	Square	Tetrahedron
$N_{CNAP}$	203.8 (24.1)	217.5 (23.2)	232.6 (32.2)	115.1 (47.6)	98.5 (40.7)	60.1 (21.2)
∫CNAP	144.8 (21.7)	157.3 (23.7)	171.8 (29.9)	68.0 (33.2)	60.1 (31.4)	30.0 (16.1)
ratio	0.71 (0.03)	0.72 (0.03)	0.73 (0.03)	0.57 (0.07)	0.59 (0.07)	0.48 (0.08)

### Appendix C.3. Ids of Final Structures

We report here the IDS of the final structures obtained from sintering simulations.
